# Patients’ and informal caregivers’ perspectives on self-management interventions for type 2 diabetes mellitus outcomes: a mixed-methods overview of 14 years of reviews

**DOI:** 10.1186/s13690-023-01153-9

**Published:** 2023-08-04

**Authors:** Ena Niño-de-Guzman Quispe, Javier Bracchiglione, Marta Ballester, Oliver Groene, Monique Heijmans, Laura Martínez García, Janneke Noordman, Carola Orrego, Claudio Rocha, Rosa Suñol, Pablo Alonso-Coello

**Affiliations:** 1 Iberoamerican Cochrane Centre, Sant Pau Biomedical Research Institute (IIB-Sant Pau), Barcelona, Spain; 2 Cancer Screening Unit, Catalan Institute of Oncology, IDIBELL, Hospitalet de Llobregat, Barcelona, Spain; 3 Department of Paediatrics, Obstetrics, Gynaecology and Preventive Medicine, Universidad Autónoma de Barcelona, Barcelona, Spain; 4 Centro de Investigación Biomédica en Red de Epidemiología y Salud (CIBERESP), Madrid, Spain; 5 Interdisciplinary Centre for Health Studies (CIESAL), Universidad de Valparaíso, Viña del Mar, Chile; 6 Universidad Autónoma de Barcelona, Barcelona, Spain; 7 Avedis Donabedian Research Institute (FAD), Barcelona, Spain; 8 Red de Investigación en Servicios de Salud en Enfermedades Crónicas (REDISSEC), Madrid, Spain; 9 OptiMedis, Hamburg, Germany; 10 University of Witten/Herdecke, Witten, Germany; 11 Netherlands Institute for Health Services Research (Nivel), Utrecht, The Netherlands

**Keywords:** Patient preference, Perspectives, Values, Self-management, Type 2 diabetes Mellitus, Overview

## Abstract

**Background:**

Self-management interventions (SMIs) are core components of high-quality care in type 2 diabetes mellitus (T2DM). We aimed to identify and summarise the scientific evidence exploring the perspectives of patients with T2DM and their informal caregivers on outcomes of SMIs, and the key themes to enhance T2DM patient-centred care.

**Methods:**

We conducted a mixed-methods overview of reviews. We searched MEDLINE, CINAHL and PsycINFO, up to June 2021 for systematic reviews (SRs) exploring the perspectives of adults with T2DM and their informal caregivers, regarding self-management. Two reviewers conducted independently study selection, data extraction and quality assessment. We estimated the degree of overlap across SRs. We performed a qualitative analysis using a thematic synthesis approach.

**Results:**

We identified 54 SRs, corresponding to 939 studies, with a slight overlap. Most SRs (47/54, 87%) were considered high quality. We developed summaries for 22 outcomes and identified six overarching themes: (1) diabetic identity; (2) accessing healthcare; (3) experience of care; (4) engagement with self-management; (5) outcomes awareness; and (6) challenges adhering to self-management. We found important variability in how patients with T2DM and their informal caregivers value critical outcomes influenced by the disease progression and several contextual factors.

**Conclusions:**

Our findings represent what matters most to patients with T2DM and their informal caregivers regarding outcomes of SMIs. Our results can facilitate the development and evaluation of SMIs, and guide decision-making in diabetes care, including the formulation of decisions and recommendations.

**Supplementary Information:**

The online version contains supplementary material available at 10.1186/s13690-023-01153-9.

## Background

Over the last few decades, the global increase in type 2 diabetes mellitus (T2DM) prevalence has become a significant economic burden to society, health systems, and patients, most directly affecting low and middle-income countries [1–3]. T2DM is associated with a reduced life expectancy, significant morbidity, and diminished quality of life [2, 4].

Self-management interventions (SMIs) represent a component of high-quality care for patients with T2DM [2]. SMIs are complex, multifaceted interventions, with increasing evidence of their beneficial effects, such as improving personal skills, knowledge, and self-efficacy, and in T2DM, a positive impact on glucose control [5–10]. However, it is still necessary to identify the most important SMI components, for each type of outcome, and under what circumstances they can be recommended and implemented.

Developing recommendations in healthcare requires a transparent step-by-step process, considering not only the effectiveness of interventions but also how patients value the importance of outcomes [11]. Patients’ preferences regarding outcomes can be reported as utility and non-utility measures [12–14]. Utilities are used to represent the strength of individuals’ preferences for different health states. Conventionally the valuations fall between 0 and 1, with 1 representing the valuation of a state of perfect health and 0 representing the valuation of death (non-existence) [15, 16]. Non-utility measures include measures explaining patients’ preferences through qualitative or quantitative methods [13]. Qualitative evidence is particularly informative regarding complex scenarios, interventions, and experiences [17, 18].

Systematic reviews (SRs) represent the most trustworthy source of evidence of patients’ values and preferences on outcomes. Over the last decade, there has been year-on-year growth of newly published articles regarding experiences or preferences in T2DM, with a corresponding increase in published SRs [19]. SRs can explore patients’ experiences with T2DM using quantitative, qualitative, or mixed-methods approaches [18, 20, 21].

A technique to summarise a large body of evidence with many SRs is to conduct a meta-review or an overview of reviews. Overviews of reviews provide broad perspectives and synthesise research fields, ideal for informing policymakers, commissioners, and providers of healthcare services [22]. They also provide insight into areas already extensively researched versus those underresearched. We considered that an overview of reviews using a mixed-methods approach, including quantitative utility-based measures and qualitative evidence, would provide a comprehensive and valid source of evidence to inform decision-making in SMIs for T2DM. Therefore, we aimed to identify and summarise available SRs exploring the perspectives of patients with T2DM and their informal caregivers, on outcomes of SMIs. This paper presents the qualitative branch of evidence and the integration of quantitative and qualitative findings.

## Methods

This study was conducted in the context of COMPAR-EU, a European project aimed to identify the most cost-effective SMIs for T2DM, among other chronic diseases [23]. We conducted a mixed-methods overview of reviews, with a three-step approach and convergent parallel analysis [21, 24–29]. The first step consisted of summarising the evidence derived from SRs informing about patients’ preferences on outcomes, using utility-based measures. In parallel, the second step consisted of summarising SRs exploring patients’ perspectives, and non-utility measures, including qualitative findings. The third step involved the integration of both sources of evidence. We have published the quantitative utility SR results elsewhere [30]. In this paper, we present results from the second and third steps. The methodological details are summarised below, and further details are available in the protocol (PROSPERO CRD42019117867) [31]. We adopted the PRISMA statement for reporting [32].

### Eligibility criteria

**Type of reviews**. SRs were defined as so if reporting: (1) a systematic search (at least in one database), (2) a list of primary studies, and (3) a description of the method of analysis. SRs could be (1) quantitative SRs, including studies with a quantitative design (e.g., surveys, cross-sectional studies); (2) qualitative evidence syntheses, including studies that applied qualitative methods (e.g., focus groups, interviews); or (3) mixed-methods reviews, including quantitative and qualitative studies that applied qualitative synthesis.

**Phenomenon of interest.** Perceptions and experiences of patients with T2DM and their informal caregivers with outcomes of T2DM either in relation to SMIs, self-management (SM), or the experience with the disease. The outcomes of interest were the 23 core outcomes set for T2DM SMIs of the COMPAR-EU project [23]; definitions are available in the Additional file 1.

**Population**. Adult patients with T2DM and their informal caregivers. The informal caregivers were considered family or friends who help patients with disease management and daily activities without payment. We included SRs that covered more than one type of population or disease if primary studies had at least 80% of adults with T2DM or if results were reported disaggregated. We excluded SRs focused only on Type 1 diabetes mellitus (T1DM), children, gestational diabetes, or healthcare professionals’ perspectives.

**Setting and language.** We did not establish geographical or setting restrictions, except those confined to inpatient care. We included studies published in English only.

## Search strategy

We searched MEDLINE (accessed through PubMed), the Cumulative Index of Nursing and Allied Health Literature (CINAHL), and PsycINFO from inception to November 2020. The search was updated monthly using each database’s alert system until June 2021. We applied a sensitive content search strategy previously published [33] and specific terms for T2DM [Additional file 2]. We limited our search to SRs by using methodological filters in each database. Other sources were the reference list of overviews identified through our search strategy and a forward citation search of selected SRs in Scopus.

### Study selection

After achieving at least 80% agreement with an initial calibration exercise (with 10% of the references), a pair of authors (JB, CRC) screened titles and abstracts, with a subsequent independent full-text assessment. Disagreements were solved by discussion or with the help of a third author (ENDG). We managed references using Endnote X9 and Rayyan.

### Data collection

We used Nvivo 12 PRO software and Excel spreadsheets to collect and analyse qualitative data. After pilot-testing the data extraction table and the coding process, two authors (ENDG, JB) extracted the predefined data, including general characteristics, methodological characteristics, settings, participants and intervention characteristics, and themes or findings. The latter was the first stage of thematic synthesis.

### Assessment of methodological quality

We applied the 11-item Joanna Briggs Institute (JBI) Critical Appraisal Checklist for Systematic Reviews and Research Syntheses [24]. One item regarding the likelihood of publication bias was removed since it only applies to SRs conducting metanalysis. After initial calibration, one author (JB) applied this checklist, and a second author validated the responses (CRC). Disagreements were solved by consensus or, if necessary, with the help of a third author (ENDG). We calculated the percentage of positive responses and classified the quality as low (0–33%), medium (34–66%), or high quality (≥ 67%) [34].

### Assessment of overlap

We measured the extent of overlap of primary studies using a citation matrix and calculated the “corrected covered area” (CCA) [35, 36]. Overlap was classified as slight if the CCA is < 5%, moderate if it is ≥ 5% and < 10%, high if it is ≥ 10% and < 15%, and very high if CCA is ≥ 15% [36].

### Data synthesis and analysis of qualitative data

We applied a thematic synthesis approach for developing fourth level descriptive and analytical themes [37]. There are four levels of interpretation in a qualitative meta-review (overview of reviews). The first-level is the participant’s interpretation of their experiences in the primary research; the second-level is the researcher’s reflections and report on the primary study; the third-level involves the synthesis of findings from studies included in a SR, and the last is the meta-review level (fourth level). In this study, we focused on the second (as reported in SRs) and third-levels of interpretation (results and discussion sections of SRs) to derive the fourth level themes. We did not return to the original studies [38].

The process consisted of (1) line-by-line coding of results and discussion sections; (2) classifying codes according to the 23 core outcomes set of COMPAR-EU (including an active search of terms using Nvivo); (3) developing fourth level descriptive themes based on semantic correspondence; (4) developing fourth level analytical themes to establish broader patterns or relationships among findings. We analysed and reported fourth level themes with different levels of detail: (1) analytical themes per outcome, including descriptive themes providing narrative accounts per each, (2) overarching analytical themes, and (3) summary, proposing inductively a plausible explanation of how patients with T2DM (or informal caregivers) perceive the importance of outcomes of SMIs. We conducted a subanalysis for two populations, informal caregivers and patients from ethnic minorities living in Western countries.

Two reviewers (ENDG, JB) developed the synthesis of descriptive and analytical themes; one independently proposed themes, and a second reviewed them independently, followed by an iterative collaborative analysis. Both reviewers discussed alternative interpretations and ensured that fourth-order levels of interpretation remained grounded in the third and second levels (as reported in SRs). A third reviewer (PAC) independently reviewed fourth level themes and coherence of the narrative accounts.

### Integrating quantitative and qualitative evidence

We used a side-by-side comparison conjoint display [25, 39] to integrate synthesized findings from SRs reporting utility-based measures (published elsewhere) with findings from SRs reporting non-utility measures (including qualitative findings), presented in this overview. The integration process included assessing whether datasets were in discordance, confirmation, or expansion; or if there were missing or less explored areas [40]. Discordance was defined as when results were inconsistent or contradictory. Confirmation occurred when findings reinforced each other. When findings from one type of data were expanded upon insights from another, this was considered expansion [40]. We synthesized and reported narratively. One reviewer independently conducted this step (ENDG), and a second reviewer cross-checked the analysis and synthesis (PAC) [21].

## Results

### Study selection

**Fig. 1:**
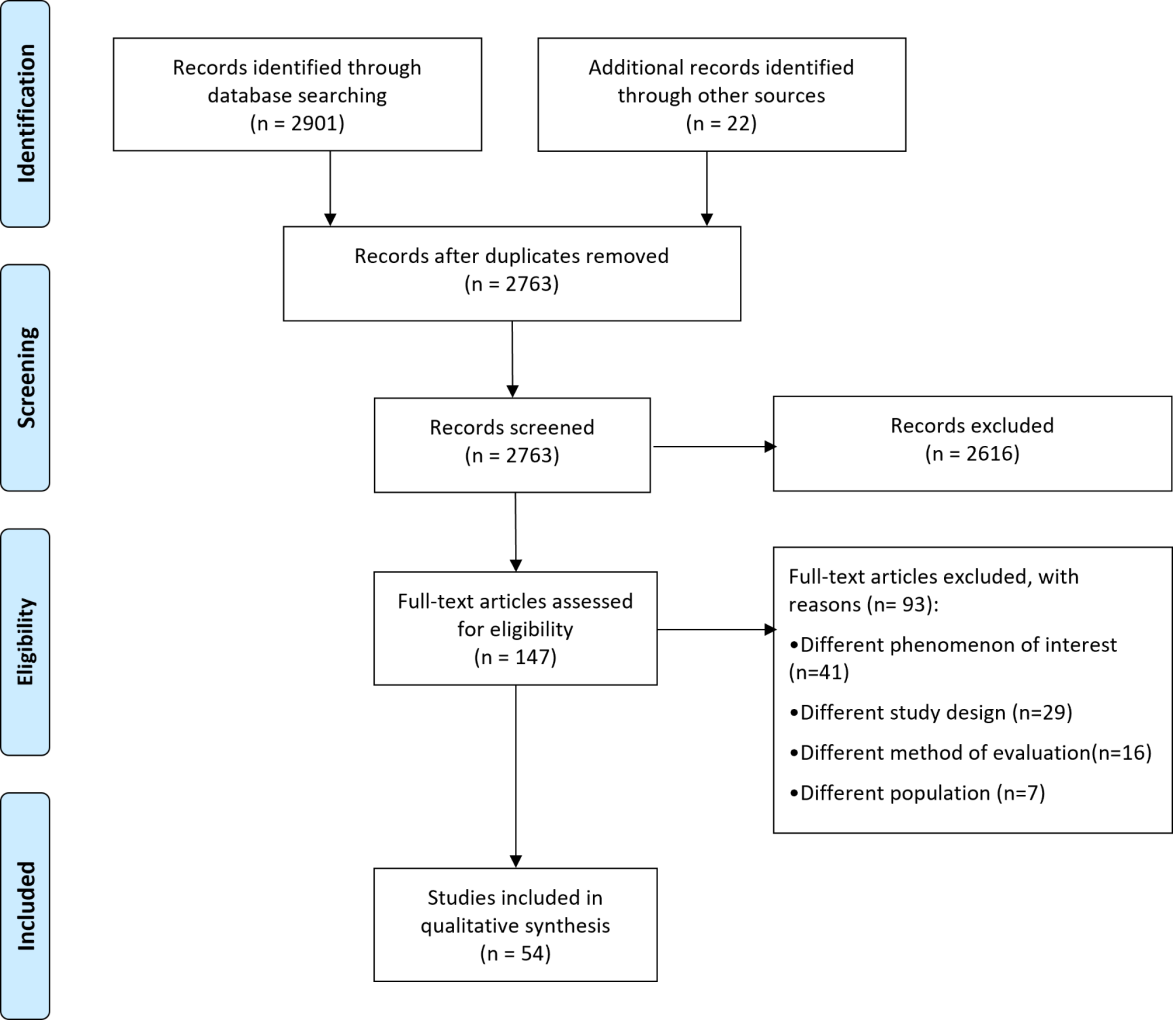
PRISMA flow diagram of study selection

We included 54 SRs, selected as described in the PRISMA flowchart (Fig. 1) (PRISMA 2020). Reasons for excluding studies reviewed in full-text are available in Additional file 3.

### Study characteristics

**Fig. 2:**
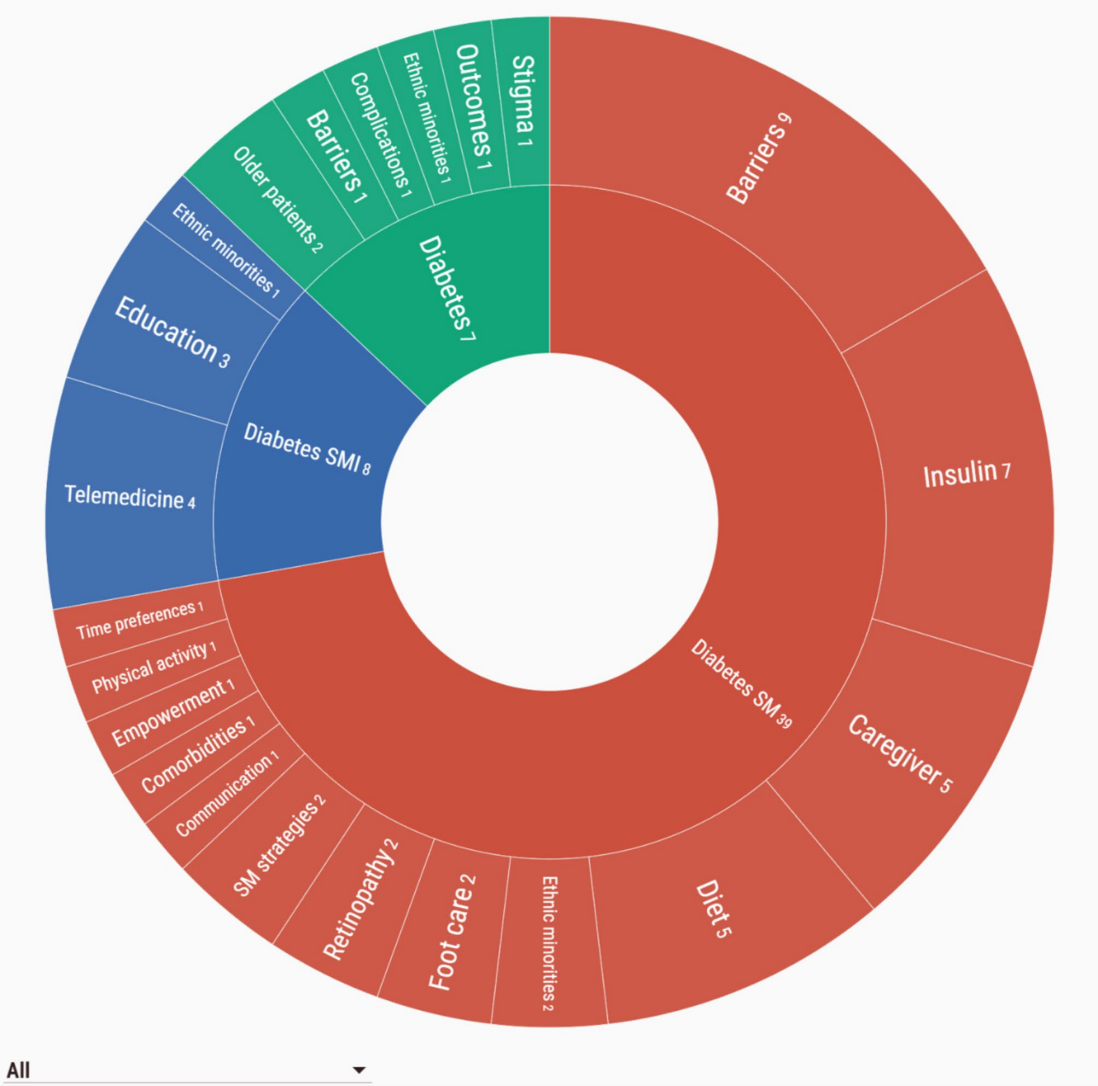
Phenomena of interest across systematic reviews Numbers represent the number of SRs in each category. **Diabetes**: SRs exploring features of the disease trajectory with broad lenses, referring to the lived experience of dealing with diabetes. It included the views of specific subpopulations, including older patients and patients from ethnic minorities. **Diabetes SM**: SRs focused on themes regarding SM behaviours, and abilities patients acquire in diabetes care. The subcategory Barriers represents SRs exploring challenges and facilitators to SM, and Caregivers, refer to SRs exploring the role of social support in SM. **Diabetes SMI**: SRs exploring perspectives and experiences with components of SMI, such as telemedicine, educational programmes, and culturally tailored SMI (in ethnic minorities)

The main characteristics of included SRs are described in Table 1 and Additional file 4. We included 25 qualitative evidence syntheses (25/54; 46.3%), 22 mixed-methods reviews (22/54; 40.7%), and seven quantitative SRs (7/54; 13.0%), corresponding to 939 primary studies. These were conducted in 19 different countries; the most frequent were UK (14/54; 25.9%), USA (8/54; 14.8%) and Australia (6/54; 11.1%). The majority were published between 2016 and 2020 (32/54; 59.3%) and included a range from 5 to 120 primary studies. Most SRs included patients with T2DM only (38/54; 70.4%), and some focused on patients from ethnic minorities living in Western countries (8/54; 14.8%), and others included informal caregivers (7/54; 11.1%). The majority addressed features of SM (39/54; 72.2%) (Fig. 2, Additional file 4).

**Table 1:** General characteristics of included reviews

*Characteristics*	*n (%)*	*References*
**Total**	54 (100)	
**Type of review**		
Qualitative evidence synthesis	25 (48.1)	[41–43, 45, 51–55, 57, 58, 60, 61, 63, 66, 68, 70, 71, 74, 80, 86, 89, 96, 97, 102]
Mixed-methods research synthesis	22 (40.7)	[44, 46–50, 56, 59, 62, 64, 65, 67, 69, 72, 73, 78, 82, 84, 87, 88, 91, 103].
Quantitative systematic review	7 (11.1)	[75–77, 81, 83, 85, 90].
**Country (contacting author)**		
UK	14 (25.9)	[45, 46, 50, 52, 56, 61, 62, 64, 65, 71, 73, 85, 86, 89].
USA	8 (14.8)	[47, 51, 55, 69, 82, 84, 90, 97].
Australia	6 (11.1)	[58, 60, 72, 75, 77, 80].
Sweden	3 (5.6)	[43, 54, 66].
Canada	3 (5.6)	[44, 57, 70].
New Zealand	3 (5.6)	[41, 49, 102].
Iran	2 (3.7)	[67, 87].
Taiwan	2 (3.7)	[59, 63].
The Netherlands	2 (3.7)	[76, 91].
Denmark	2 (3.7)	[78, 81].
Other	9 (16.7)	[42, 48, 53, 68, 74, 83, 88, 96, 103].
**Publication year**		
2006–2010	5 (9.3)	[44, 48, 54, 61, 76].
2011–2015	17 (31.5)	[45–47, 50, 51, 59, 63, 70, 72, 75, 80, 83–85, 87, 88, 90].
2016–2020	32 (59.3)	[41–43, 49, 52, 53, 55–58, 60, 62, 64–69, 71, 73, 77, 78, 81, 82, 86, 89, 91, 96, 97, 102, 103].
**Number of included studies**		
5–20	30 (55.6)	[44, 48, 52, 54, 55, 59–66, 68–70, 72, 74, 75, 77, 78, 80, 81, 83, 85, 87, 89, 90, 97, 102].
21–40	18 (33.3)	[41, 43, 45, 49–51, 56, 58, 67, 71, 76, 82, 86, 88, 91, 96, 103].
42–120	6 (11.1)	[46, 47, 53, 57, 73, 84].
**Population of interest**		
Only T2DM	38 (70.4)	[41–43, 45–47, 49, 52, 55–60, 62, 65–71, 74, 75, 77, 78, 80, 82–84, 86–89, 91, 96, 97, 102].
T1DM and T2DM	16 (29.6)	[44, 48, 50, 51, 53, 54, 61, 63, 64, 72, 73, 76, 81, 85, 90, 103].
Ethnic minorities in Western countries	8 (14.8)	[41, 46, 50, 61, 68–71].
Patients and informal caregivers	7 (13.0)	[42, 58, 60, 78, 82, 84, 89].
**Phenomenon of interest**		
T2DM SM	39 (72.2)	[41, 42, 44–51, 53–60, 63, 64, 67, 69–73, 78, 81–85, 87–90, 96, 97].
Self-management interventions	8 (14.8)	[62, 68, 74–77, 102, 103].
T2DM	7 (11.1)	[43, 61, 65, 66, 80, 86, 91].
**Quality assessment tool**		
CASP	18 (33.3)	[41, 49, 52, 53, 56, 58–60, 65, 66, 68, 70, 72–74, 78, 86, 96].
JBI-QARI	5 (9.3)	[51, 63, 67, 80, 97].
Other **	9 (16.7)	[49, 50, 55, 64, 69, 72, 76, 88, 103].
Not specific or adapted versions	11 (20.4)	[42, 44–46, 81, 85, 87, 89–91, 102].
Not assessed	12 (22.2)	[43, 47, 48, 54, 57, 61, 62, 71, 75, 77, 82, 84].

*Other countries: Malaysia, Ireland, Germany, Thailand, Norway, China, Saudi Arabia, Korea, Singapore

JBI-QARI (Joanna Briggs Institute Qualitative Assessment and Review Instrument), CASP (Critical Appraisal Skill Programme)

**STROBE statement, McMaster University’s Guidelines for Qualitative Review, Pluye’s mixed methods appraisal tool, qualitative studies using Popay et al. and Jadad et al. and Creswell and Plano Clark, QualSyst, NICE checklist, Mixed Methods Appraisal Tool

### Studies’ quality and overlapping

Most SRs evaluated the methodological quality of included primary studies (42/54; 77.8%) using different tools. The most frequent was the Critical Appraisal Skill Program checklist (18/54; 33.3%). One study applied CERQUAL to assess the certainty of evidence [41]. When evaluating the SRs’ quality with the JBI Critical Appraisal Checklist, most were considered high quality, with ≥ 67% (47/54; 87%). The most frequent concern was the absence or unclear criteria to appraise studies (23/54; 42.6%) (Additional file 5). The overlap analysis of the primary studies showed a corrected covered area of 0.75%, meaning there was a slight overlap. When comparing SRs, we identified that 126 studies were included in at least 2 SRs, 40 in 3 SRs, and 20 in 4 SRs (Additional file 6).

### Fourth level themes

We developed summaries, including descriptive and analytical themes with narrative accounts for all the outcomes of SMI for T2DM from the core outcome set of COMPAR-EU, except for *unscheduled care.* Summaries per outcome are available online. See: https://osf.io/dj3wy/?view_only=f0f3a82ea97747b59beb4de2a11c05f8. We identified 101 descriptive themes (Table 2) and six overarching analytical themes; their narrative accounts are described below.

**Table 2:** Fourth level descriptive themes per outcomes of Self-Management interventions

*Outcomes of SMIs*	*Descriptive themes*	*References*
***Knowledge***	Insufficient knowledge influences self-management (SM) behaviours	[44, 46, 48, 50, 51, 53, 59, 66, 70, 71, 73, 83].
	Knowledge can improve well-being but does not necessarily influence SM behaviours	[44, 47, 49, 57, 59, 74].
	Information needs and training preferences are variable	[43, 46, 53, 54, 57, 60, 66, 68, 70, 74, 84, 89, 103].
	Traditional health beliefs may explain patients’ understanding of diabetes	[42, 46, 50, 61].
***Health literacy***	Health literacy allows making informed decisions and accessing health services	[47, 49, 50, 57, 61, 62].
	Cognitive representations of the disease determine patients’ SM	[53, 63–65)
	Patients’ and healthcare professionals’ perspectives on the provision of information are divergent	[45, 46, 60, 65].
***Self-efficacy***	Low self-efficacy can inhibit self-care leading to a spiral downwards situation	[45, 53, 55].
	Tradition and culture influence self-efficacy	[41, 46, 50, 61, 70].
	Adequate support and information help to achieve empowerment	[45, 82].
	Acknowledgement of patients’ characteristics, diabetes psychological and emotional burden, chronicity of the disease, and treatment complexity may help to increase self-efficacy	[51, 68].
	Developing self-efficacy skills can be challenging but, when achieved, enhances self-care	[42–44, 53, 55, 85].
	Patients with intellectual disabilities and their informal caregivers can gain self-efficacy with flexible and creative support strategies	[89].
***Patients’ activation***	Active SM requires adopting a diabetic identity taking responsibility, and control	[42–44)
	Self-care ability develops within the tension between disease and life-centred approaches	[44, 51].
	Patients face the challenge of modulating between living in the present and the future	[42, 43].
	A low activation level is predicted by personal models or perceived barriers	[44, 48, 52, 54, 56, 57, 59].
	Lack of resources and having a passive role in decision-making hinder patient activation	[42, 44, 47, 51, 58].
***Adherence to a healthy diet***	Abstract Knowledge and practical understanding are crucial for dietary modification	[42, 46, 57, 61, 70, 83, 84].
	Healthy diet adherence is challenging and requires self-discipline and proactivity	[41, 43, 46, 57, 61, 66, 85, 86].
	Patients’ social contextual factors can act as barriers or facilitators of adherence to a healthy diet	[41, 43, 46, 49, 58, 66, 68, 70, 71, 78, 82].
	Tradition over the significance of food may limit dietary modifications	[41, 46, 61, 69, 70].
	Western dieting advice contrasts with cultural paradigms of ethnic minorities	[41, 46, 61, 70].
	Fear of acquiring the “sick identity” is a barrier to dietary changes	[43, 50].
***Physical activity***	Patient’s motivation to engage and persist in physical activity is delineated by the expected or experienced benefits rather than Knowledge	[49, 55, 70].
	Physical activity persistence requires self-efficacy	[49, 55, 70].
	Perceived support influences on patients’ confidence to engage in physical activity	[55, 58, 68, 73)]
	Physical impairments usually affect patients’ self-care ability representing barriers to physical activity	[44, 46, 55].
	Psychological barriers to physical activity include negative attitudes and negative feelings towards exercise	[48, 51, 55, 70, 86].
	Financial constraints and living in an impoverished environment are barriers to physical activity	[44, 46, 70].
	Cultural barriers, including beliefs, gender norms, and social rules, can stop patients from engaging in physical exercise	[46, 50, 61, 70, 83].
	Environmental factors can limit physical activity	[46, 55, 70].
***Adherence to treatment***	Medication prescription usually generates negative emotions. However, positive medication experiences can reinforce positive perceptions of medication effects	[52].
	Medication adherence requires self-regulation and deliberate effort	[52].
	Patients prefer simplified regimens, which can enhance adherence to the treatment	[47, 48, 52, 71, 85, 87].
	Patients worry about the perceived medication effectiveness and the risk of adverse events	[52, 70, 71].
	Patients can present five patterns of medication-taking behaviours	[52, 85].
	Most patients attribute negative features to insulin therapy	[46, 52, 59, 88].
	Factors that influence medication-taking behaviours and adherence to treatment are diverse	[46, 47, 50, 56, 58, 59, 61, 70, 71, 78, 83, 85, 87, 88].
	Fear of injection, pain and inconvenience of insulin administration are significant barriers to treatment progression to insulin	[46, 52, 56, 59, 85, 90].
	Fear of hypoglycaemia and weight gain are associated with reduced adherence to insulin therapy	[52, 56, 59, 90].
	Psychosocial factors and stigma can hinder adherence to insulin therapy	[46, 52, 56, 59, 67, 85, 88, 89].
	Motivators of compliance with treatment include fear of complications, having comorbidities, awareness of the need for injections, receiving support and having a lower perceived burden	[44, 45, 49, 52, 56, 59, 88, 90].
***Self-monitoring***	Self-monitoring of blood glucose (SMBG) improves the awareness of the state of health	[43, 46, 47, 59, 63, 66].
	Disparate perceptions between patients and HCPs can decrease the efficacy of SMBG	[45, 63].
	Barriers to SMBG include misconceptions, problems operating the device, test discomfort, interruption in daily life and a need for family support	[44, 58, 63, 66, 78, 84].
	Insulin use influences behaviours toward the monitoring results	[56, 63].
	Patients’ responses to self-monitoring are diverse and personal; not all patients are active problem solvers	[56, 63, 75, 86].
**(** ***Other) SM behaviours***	SM is connected to everyday life where patients’ network is a functional part	[41, 42, 44, 58, 61, 78, 80, 81].
	Self-care is defined as an evolving development process that facilitates an optimal self-management	[44].
	Context shapes self-management behaviours	[42, 45–50].
	Establishing a mutual relationship with HCPs is crucial for enhancing SM behaviours	[42–45, 48, 60].
	Having education or training, family support and a great sense of responsibility facilitate engagement in foot self-care	[53, 72].
	Patient’s willingness to adopt technology is influenced by independence, perceived improvement in the quality of life and ease of use	[75, 76].
***Glycemic control***	Negative behaviours and fatalistic beliefs are associated with poor glycaemic control	[50, 54, 81].
	Knowing HbA1c values leads to a better understanding of diabetes SM. However, it is not enough to increase confidence and motivation to perform SM activities	[47].
***Weight change***	Weight concerns influence adherence to treatment	[52, 56, 90].
	Social support and patient-HCP interaction have mixed results in weight management	[47, 52, 60].
***Competing comorbidities*** Originally this outcome was ***Blood pressure***; we extended it to comorbidities.	Simultaneous demands of competing comorbidities, such as back pain, arthritis, asthma, congestive heart failure, chronic obstructive pulmonary disease, fatigue, depression, hypertension and cancer, represent an extra burden for patients and barriers to self-management	[44, 47, 49, 85, 88].
***Lipid control***	Some patients do not perceive the benefit of taking lipid-lowering medicines	[71].
	Some patients are not aware of their increased cardiovascular disease risk	[65].
***Long-term complications***	Barriers and enablers to retinopathy screening are multi-dimensional, mainly related to environmental context and resources; social influences; Knowledge; memory, attention, and decision processes; beliefs about consequences; and emotions	[64, 73].
	Patients’ comprehension of diabetic foot ulceration is often limited or erroneous	[53, 72, 76, 77].
	Factors determining foot self-care include specific barriers and facilitators, views over therapeutic footwear, and patients’ attitudes toward taking risks	[53].
	Most patients are not satisfied with their foot-related healthcare experiences and demand more holistic care	[53].
	The process of discovering and seeking treatment for an ulcer can be prolonged	[53].
	Diabetic foot ulceration has a wide-ranging impact on patients’ life	[53].
	Telehealth use for diabetic complications improves patient-HCP interactions in self-care	[76, 77].
	Self-estimation of risk of cardiovascular complications is variable, with most patients having a low-risk perception	[65].
***Hyperglycaemia***	Patients do not always acknowledge a causal relationship between hyperglycaemia and symptoms or complications	[46].
	Hyperglycaemia is a reason for psychological and emotional distress	[47, 54, 66, 83].
***Hypoglycaemia***	Hypoglycaemia detection is challenging and may have a disrupting effect on patients’ life	[43, 56, 78].
	Fear of hypoglycaemia impacts patients’ SM	[46, 52, 54, 57, 59, 66, 85, 86, 88].
	Family support is highly valued in hypoglycaemic events	[58, 78, 84].
***Death / Life expectancy***	Fatalism is associated with a sense of hopelessness and powerlessness	[41, 50, 53, 56, 65, 70, 71, 86].
	Patients have mixed perceptions about the relationship between medicines and death	[59, 71, 88].
***Quality of life***	A myriad of emotions is related to diabetes diagnosis, experiencing complications and the complexity of SM	[43, 46, 53, 56, 57, 73, 86, 89].
	Diabetes threatens how patients identify themselves (their “sense of identity”)	[43, 45, 46, 53, 80].
	Social support is crucial to SM, but in some cases, it can be perceived as an interference	[44, 46–49, 53–55, 57, 58, 70, 73, 78, 80, 84, 88].
	Stigma related to diabetes diagnosis, treatment and complications is frequent and hinders SM	[41, 46, 56, 71, 73, 85, 89, 91].
***Experience of care***	Patients prefer individualised care over a generic one	[43, 45, 49, 60, 63, 66, 74].
	The patient-healthcare professional relationship can be paternalistic or collaborative	[46, 51, 53, 60, 66, 67].
	Despite valuing emphatic interactions, patients usually face criticism and blame for them	[44, 53, 54, 60, 67].
	Cultural appropriateness shapes the experience of care	[46, 50, 54, 67–70].
	Patients are usually willing to use technology	[74, 75]
	Patients are usually satisfied with the adoption of technology	[76]
***Decision-making process***	Patients and HCPs have a divergent agenda	[42, 56, 60]
	The quality of communication influences the decision-making process	[53, 60, 67, 71, 72).
	Culture shapes preferences for the decision-making process	[54, 67, 70]
	Lack of time is a common issue affecting patient-professional communication	[42, 53, 54, 60, 67, 70]
***Scheduled care***	Difficulties getting to the appointment	[49, 61, 70]
	If patients do not feel heard, they might not return	[60, 66]
	The cultural background might be a reason for delaying consultations	[61, 71]
	The experiences with primary health care professionals influence attendance to specialist appointments	[42, 64, 73]
	The process of accessing specialised care is perceived as difficult	[64, 73]
	Access to specialised care is influenced by setting and cultural background	(73).
***Value for money of SM***	Type of insurance influences diabetes treatment	[41, 42, 47, 53, 62, 71, 85]
	Diabetes may have an impact on patients’ and informal caregivers’ finances	[56, 84]
	Patients’ finances may have an impact on disease management	[41, 42, 44, 47–49, 61, 70, 72, 76, 77, 83, 88]
	Telehealth care is perceived positively from a financial point of view	[56, 84]

### Diabetic identity

Patients with T2DM need to adopt a “diabetic identity” to confront the diagnosis and have a sense of ownership in disease management. However, not all patients are willing or able to SM [42–44]. Adopting a diabetic identity is fundamental to effective SM. The ability to engage in diabetes care (“sense of agency”) is determined by several contextual factors, of which financial ability is central [42, 45–50]. Self-care ability evolves in the tension between disease taking control of life, and patients taking control of the disease and living in the present, and for the future [44, 51]. Patients can start their T2DM journey with a medication prescription, associated with negative emotions. Their social environment, beliefs and experiences can reinforce their perceptions [52]. Talking to peers and sharing experiences is an important source of emotional support [53, 54]. These interactions provide opportunities to discuss challenges and fears openly and a sense of belonging to a group, which enhances confidence to overcome the barriers to initiate and maintain SM [55].

Beliefs about the seriousness of diabetes, treatment effectiveness and a passive role in interacting with healthcare providers (HCPs) predict the level of SM in diet, exercise, and glucose testing [42, 44, 47, 48, 51, 52, 54, 56–59]. Good communication and effective collaboration between patients and HCPs are crucial to generate opportunities for self-care knowledge, facilitated by regular, repeated and timely contact [42–45, 48, 60].

### Accessing healthcare

Patients need access to and interaction with healthcare services and HCPs, to make informed decisions. Low numeracy, literacy skills or health literacy impede patients from understanding health-related information and receiving or accessing diabetes care services [47, 50, 57]. Challenges to gaining knowledge and finding information are higher in patients who do not speak or read the dominant language fluently [50, 61, 62]. Patients with inaccurate cognitive disease representations have alternative interpretations or biased perceptions of their risk of complications [45, 53, 63–65]. Preferences for the amount and quality of the information provided by HCPs are mixed. Some patients are satisfied with the information, others ask for more pragmatic advice, and others feel reluctant or unable to articulate their concerns [45, 60]. While HCPs of patients with low health literacy seek to improve knowledge by repeatedly checking for comprehension, patients value being heard and asked about their circumstances, making the information more relevant [65].

### Experience of care

The experience of care depends on the degree of individualised care, the nature and characteristics of the patient-HCP relationship, and cultural appropriateness. Most patients value an individualised provision of care, feeling heard and being given recommendations, tailored to their perceived needs [43, 45, 49, 60, 63, 66]. Patients identify that relationships with HCPs can be collaborative, sharing control and responsibility, or paternalistic, where HCPs are an authority [46, 51, 53, 60, 66, 67]. Cultural appropriateness of care includes linguistic appropriateness and awareness of the mismatch between recommendations and patients’ beliefs [46, 50, 54, 67–70].

The divergent agenda between patients and HCPs, the quality of communication, the cultural context, and the lack of time, influence the decision-making process in diabetes care. Patients value emphatic interactions. However, they usually face criticism and blame, which undermines communication and autonomy [44, 53, 54, 60, 67]. High-quality communication defined as the effective and meaningful exchange of information that considers patients’ unique requirements, values, and preferences, is associated with improved SM and enhanced well-being [53, 60, 67, 71, 72]. Patients perceive a lack of time as inadequate support [42, 53, 54, 60, 67, 70]. Patients who feel unheard do not attend appointments, avoid asking questions, or discuss personal issues [60]. Patients perceive HCPs as disease-oriented, leaving patients feeling that their experiences are devalued, discouraging SM efforts [42, 56, 60]. Trustful and friendly communication with HCPs facilitates participation, responsibility, and safety in self-care [66].

### Screening for complications

Receiving a recommendation from primary care HCPs facilitates retinopathy screening [64, 73]; this decision is influenced by the perception of how competent or qualified the HCPs are [73]. Patients often report problems with referrals, scheduling appointments, long waiting times and lengthy appointments, which can be problematic because of food abstinence [64, 73]. Barriers to primary care or retinopathy screening clinics include transportation, language and cultural barriers, and work commitments [42, 49, 70, 73]. Although most patients prefer flexibility when setting appointments, some value fixed ones [73]. Most patients are willing to use and are generally satisfied with technological health interventions [74, 75]. The willingness to adopt them is influenced by the sense of independence, the perceived improvement and ease of use [75, 76]; however, some perceive it affects trust and confidentiality with HCPs [76].

Barriers to attending retinopathy screening and foot care are multi-dimensional [53, 64, 73]. Patients usually have limited comprehension of diabetic foot ulceration (DFU) and amputation, which have significant and enduring effects on patients’ quality of life. Overall, most patients ignore foot care advice to maintain a normal life [53]. Telehealth interventions for foot care could improve patient-HCP interactions [76, 77].

### Engagement with self-management

#### Self-efficacy

Patients’ reflections on SM are connected to everyday life habits, traditions, cultural beliefs, preferences, attitudes, and the patient’s social network [42, 44, 46, 50, 58, 61, 70, 78–81]. Developing self-efficacy skills can be challenging, especially when patients perceive having full responsibility, limited control, a sense of hopelessness and resignation [42–45, 53, 55]. Patients become more proactive when they start achieving goals, feel in control, understand their responsibility, and gain confidence [44, 53]. Patients consider SM support adequate when they receive timely information and advice, and perceive HCPs have considered their circumstances, the psychological and emotional burden of T2DM, disease chronicity, and treatment complexity [45, 51, 68, 82]. The burden of prescribed self-care and unrealistic expectations of HCPs are barriers to SM compliance [44].

### Healthy diet

Most patients and informal caregivers recognise having limited knowledge regarding nutritional concepts and how to implement them [42, 46, 57, 61, 70, 83, 84], and perceptions of a healthy diet are culturally influenced [46, 61, 69, 70, 79]. Adopting a healthy diet requires self-discipline strategies, such as portion control, avoiding tempting food and being proactive. However, healthy options are often reported as unreachable due to their high costs [41, 46, 49, 58, 61, 66, 68, 70, 71, 85]. Social situations and family support influence adherence to a healthy diet; some patients may need to adjust and subordinate their diet due to social factors [41, 43, 46, 49, 58, 66, 68, 70, 71, 78, 82].

### Physical activity

Knowledge is insufficient to initiate and engage in physical activity; patients need physical and non-physical skills [55]. Expected and experienced benefits influence these decisions [49, 55, 70]. Barriers to physical activity include low self-efficacy, negative attitudes, physical weakness, symptoms persistence, comorbidities, physical limitations, financial constraints, unsafe neighbourhoods, competing demands, perceived social support, travel distance, lack of culturally sensitive options and transport [44, 46, 48, 50, 51, 55, 58, 70, 73, 83, 86].

- **Treatment and medicine-taking behaviour**.

Adherence to treatment and lifestyle behaviours requires self-regulation, and a deliberate effort to live as normally as possible [48, 52, 85, 87]. Patients can present five patterns of medicine-taking behaviour: (1) strict adherents who strongly believe in treatment benefits; (2) those who accidentally miss a dose, who usually feel guilty; (3) unintentional non-adherents who do not feel guilty, possibly due to lack of symptoms and a belief that diabetes is not serious; (4) intentional non-adherents who delay, skip, or adjust doses, often manipulating blood glucose and diet, without feeling guilty, and (5) intentional non-adherents who feel guilty and usually had negative experiences with treatment [52]. Most patients wish to minimise daily medications or simplify their regimens [47, 52, 71]. Factors influencing treatment adherence are multi-dimensional [46, 47, 50, 56, 58, 59, 61, 70, 71, 78, 83, 85, 87, 88]. Patients’ worries include the treatment’s effectiveness, side effects (hypoglycaemia or weight gain), interactions between different medication regimens that could have negative long-term effects, needle anxiety, fear of injection, pain, stigma and discrimination [52, 70, 71]. Insulin is perceived as an indicator of the worse type of diabetes and is associated with more side effects than oral glucose-lowering agents. Insulin therapy is considered inaccessible, impractical and unacceptable, restricting patients’ lives, including daily and social activities [46, 52, 56, 59, 85, 88, 89]. The complexities of managing insulin include injection difficulties and regimen inflexibility, forgetting doses, and the titration of the insulin dose [46, 52, 56, 59, 85, 90]. Fear of complications, experiencing comorbidities, awareness of treatment necessity, receiving support and temporary trials, reduce barriers to treatment progression to insulin [44, 49, 52, 59, 71, 88, 90]. Patients prefer non-judgmental guidance on alleviating negative experiences with insulin, having some control over changes and real-life advice [45, 52, 56].

#### Self-monitoring

Self-monitoring of blood glucose (SMBG) helps patients understand the relationship between blood glucose levels and disease progression, treatment and prognosis. However, despite knowing what to do, patients might not always have the time or energy to respond [43, 46, 47, 59, 63, 66]. Patients prefer when HCPs customise blood glucose plans based on their condition; however, inadequate HCPs interactions decrease the willingness and efficacy of SMBG [45, 63]. Barriers to SMBG include problems with monitoring devices, lack of confidence, misconceptions, fatalistic beliefs, and the perceived burden [44, 58, 63, 66, 78, 84]. Patients not using insulin focus on regulating daily food intake and lifestyle, while patients who use insulin focus on insulin dosage. SMBG has become a helpful habit for preventing and detecting hypo- and hyperglycaemic symptoms [56, 63]. Some patients who perform SMBG refuse to assume an active role, while others are willing to accept the responsibility actively [56, 63, 75, 86]. Foot self-care is often considered a lower priority than immediate demands [53, 72].

### Outcomes awareness

Poor glycaemic control is frequently associated with negative behaviours, impatience, and fatalistic beliefs, or being just aware of their HbA1c values but not feeling confident and motivated to improve their diabetes [47, 50, 81]. Some patients develop incorrect causal relationships between symptoms and complications, considering symptoms of poor SM and complications inevitable [46]. Patients often deal with psychological and emotional distress or a sense of failure due to unacceptable blood sugar levels and the constant threat of hypo- and hyperglycaemia [47, 54, 66, 83].

Most patients do not experience warning signs of hypoglycaemia. Its ocurrence causes fear and reduced treatment adherence, which explains why some patients prefer maintaining high blood glucose levels [46, 52, 54, 56, 57, 59, 66, 85, 86, 88]. Insulin-related nocturnal hypoglycaemia disrupts diabetes SM and quality of life [43, 56, 78]. Weight and lipid control are SM goals. Fear of weight gain or the inability to lose weight is a common worry for patients using insulin; however, for others, weight loss can be an expected benefit [52, 56, 90].

Social support and interactions with HCPs influence adherence to weight loss educational programmes [47, 52, 60]. Patients often do not perceive the benefit of lipid control [71], probably due to the absence of symptoms and the lack of awareness of the increased cardiovascular risk [65].

#### Challenges adhering to self-management

Barriers to SM include insufficient knowledge about the seriousness of the disease, the risk of complications or the importance of preventive care [44, 48, 51]. Patients’ beliefs regarding diabetes causes and the perceived sense of control influence SM behaviours [42, 46, 50, 61]. Even though knowledge can improve well-being, it is not enough to motivate patients to engage in healthy behaviours. The lack of motivation is the main obstacle to seeking information. Some patients feel it is pointless to manage the disease since complications would manifest regardless of any action taken [47, 49, 59, 74].

Patients often feel overwhelming negative emotions related to diabetes diagnosis, complications, and SM [46, 56, 57, 73, 80, 86]; they experience a loss of confidence, the disruption of usual roles and their sense of independence [43, 45, 46, 53, 56, 57, 73, 80, 86, 89]. The stigma associated with T2DM, defined as patients’ expressions of embarrassment and moral failure, can be related to diagnosis, complications, and medication use. Stigma may prevent patients from disclosing their diagnosis, leading to an impaired ability to SM [41, 46, 53, 56, 71, 73, 85, 89, 91]. Simultaneous demands of competing comorbidities represent an extra burden [44, 47, 49, 85, 88].

Patients usually feel worried or anxious about premature death, despite underestimating the likelihood of fatal events [65, 86]. Some patients express hopelessness, powerlessness, a sense of inevitability, and fatalism regarding diabetes, irrespective of treatment. They consider decisions in the past cannot be redressed [41, 50, 53, 56, 71]. The sense of fatalism leads to low patient motivation to partner HCPs, which could be influenced by culture.

Patients’ finances have an impact on disease management. Patients experience a continuous trade-off between health-related costs and other concerns [41, 42, 44, 47–49, 61, 70, 72, 76, 77, 83, 88]. Patients with acute or chronic complications report more significant healthcare expenditures. Diabetes can considerably impact patients’ and informal caregivers’ finances [56, 84]. Insurance schemes with reduced or non-existent co-payments improve treatment adherence. Some patients perceive the limited availability of personnel and resources due to the need for more healthcare system funding [41, 42, 47, 53, 62, 71, 85]. Patients and informal caregivers perceive telehealth care as alleviating financial burdens due to reduced healthcare utilisation and lower treatment and travel costs [42, 76, 77]. The economic impact of medical expenses on patients and informal caregivers was most frequently reported in high-income countries without public health system funding. There is a significant disparity in the financial burden for vulnerable subpopulations, such as immigrants, older people, and patients with lower levels of education; in some cases, cultural factors may also play a role.

#### Subgroup analysis

##### Family informal caregivers

Family informal caregivers face the dilemma of protection versus enabling autonomy [89]. Informal caregivers often support patients with depressive and behavioural problems. Nevertheless, they report lacking support when dealing with events that could make it difficult for patients to follow SM behaviours [84]. Patients often need family support for privacy and shared responsibility when checking blood glucose [58, 78]. Fluctuating blood glucose levels are a serious concern to informal caregivers; for instance, hypoglycaemia is challenging since they may need to take control in acute situations [58, 78, 84]. Family informal caregivers can promote dietary adherence and physical activity. Encouragement with a gentle, positive approach is well-received and effective [82]. Family is usually perceived as a source of motivation and confidence [55, 68]. However, for some patients, it is perceived as interference. Supportive interactions occur when the goal is to adhere to the recommended lifestyle, to achieve physical and psychological well-being for both of them. Non-supportive actions include sabotaging diet and promoting unhealthy family habits or routines [44, 46–49, 54, 55, 58, 70, 73, 78, 88]. Some informal caregivers fear being affected in their job and financial status due to diabetes demands [56, 84].

### Ethnic minorities living in Western countries

Patients from some ethnic minorities living in Western countries often report inevitability and fatalism in their diabetes perception, considering disease management beyond their control or a consequence of their past decisions. However, some perceive faith has a direct influence on the outcome but does not absolve them from taking responsibility [41, 46, 50, 61]. A sense of fatalism leads to low motivation to partner with HCPs to address diabetes management [41, 50, 70, 71]. The cultural significance of food usually conflicts with the Western concept of a healthy diet, which is portrayed as a form of self-denial to improve health. These patients often describe strong food traditions, despite awareness of the detrimental effects of some types of food or behaviours on health, and the importance of following a healthy diet. When HCPs advise against some traditional foods and discount them as harmful, it can be difficult to follow dietary recommendations, preventing change and SM [41, 43, 46, 50, 61, 70]. Patients are often unaware of the risks of experiencing complications, especially macrovascular events [65].

Most South Asians in Western countries prefer traditional therapies and lay sources of knowledge. They perceive that exercise depletes energy and prioritises the family’s diet over their needs; they also perceive that diabetes care burdens their family [61, 71]. The main factors affecting the experience of care are linguistic appropriateness, and the mismatch between HCPs’ recommendations and patients’ beliefs [46, 50, 54, 67, 70].

Middle Eastern or South Asia cultures rely more on tradition and authority, making it challenging to engage in shared decision-making [54, 67, 70]. Most patients consider physicians the primary information source and acknowledge low self-efficacy [42, 46, 50, 61, 70]. Patients value linguistically concordant support highly [50, 69]; however, some feel reluctant to communicate with HCPs through interpreters [46, 50, 70]. Most patients prefer professional interpreters to relatives or friends [50, 70]. Participants of culturally tailored interventions consider that these interventions facilitate healthcare access [68].

### Integrating utility and non-utility measures for outcomes of T2DM-SMIs

We developed a conjoint display integrating quantitative (utility-based measures) and qualitative findings (non-utilities measures) (Table 3). For glycaemic control, results from both sources of evidence expanded each other; however, some discordance was identified in poor control that mismatched qualitative findings. Evidence from utility measures was informed by a single study, and would require additional research to confirm or not this value.

**Table 3:** Conjoint display utility and non-utility measures of outcomes importance

Outcomes	Utility* measures	Non-utility measures	Mixed-methods findings
***HbA1c (glycaemic control)***	Glycaemic control is an expected treatment outcome, usually preferred over avoiding hypoglycaemic events (104). Achieving glycaemic control obtained a high overall willingness to pay despite some variability within and across studies (28 to $205 US/month across) reported in five studies conducted in Europe, USA and Australia, included in a systematic review (104). Utilities showed no differences when comparing poor control 0.85 (95% CI 0.80 to 0.90) vs. excellent control 0.87 (95% CI 0.82 to 0.92) (EQ-5D) in a study conducted in Japan [105, 106].	Poor glycaemic control is frequently associated with negative behaviours, impatience, and fatalistic beliefs or being just aware of their HbA1c values but not feeling confident and motivated to improve their diabetes [26, 29, 60]. Patient reactions to self-monitoring blood glucose (SMBG) results vary and are often subjective. While some patients have few problems, others see SMBG as a burden that has a significant impact on their lives, causes anxiety, and leads to numerous internal and external psychological conflicts [56, 63, 86].	***Expansion /discordance*** Overall, both sources of evidence complement each other. Quantitative findings describe the desirability of achieving glycaemic control, while qualitative findings describe the experience of not achieving glycaemic control and the burden associated with self-monitoring. However, contradictory findings were described for poor control. Unexpected findings in one study conducted in Japan, showed similar impact on health for poor and excellent control.
***Weight change***	The impact of extreme obesity on health is twice as important as being overweight. Extreme obesity has a mean utility value of 0.400 (95% CI 0.363 to 0.437), while obesity and overweight have 0.8 (EQ-5D) [105, 107, 108]	Fears of weight gain or the inability to lose it was a common worry that mediated insulin adherence [52, 56, 90). Weight loss was an expected benefit of adherence to treatment for some patients (52).	***Expansion*** Weight changes concerns in utility measures is described as the impact of the severity of obesity, whereas in the qualitative findings it is linked to the effect of treatment.
***Long-term complications***	The most important outcomes expressed by utility values were long-term complications, including diabetic peripheral neuropathic pain (0.468, 95% CI 0.372 to 0.565) [105, 107, 109), blindness (0.529, 95% CI 0.393 to 0.665) [105, 110], and amputation (0.537, 95% CI 0.453 to 0.621) [105, 107, 111]. *Cardiovascular risk* When patients were asked about the importance of the effects of therapy on the risk of cardiovascular disease, some patients assigned a high, but not primary, importance, to not experiencing a heart attack episode within the next year. Others, consider the reduction of cardiovascular risk, in general, to be of minor importance (104).	Patients’ estimation of the risk of long-term complications is variable. Patients usually have limited or erroneous comprehension of diabetic foot ulceration and amputation [53]. *Cardiovascular risk* Patients tend to underestimate cardiovascular risk [65].	***Expansion/Confirmation*** Quantitative utility-based measures provided more detailed and extensive findings regarding long-term complications than qualitative findings, which were scarce and generic. For cardiovascular risk, findings tend to be consistent, highlighting low-risk perception.
***SM burden***	Diet and exercise reported the highest utility values (0.765, 95% CI: 0.684 to 0.846, I2: 93.9%). Results were similar for intensive blood glucose control and usual care (0.737, 95% CI: 0.640 to 0.833; and 0.737, 95% CI 0.677 to 0.798, respectively).	Adherence to SM requires training and time to integrate into everyday life and adjust to contextual factors. Accessing healthcare can be difficult in some contexts. Attendance to clinical appointments varies according to previous experiences. Most patients experience constraints on quality of life and physical and psychological barriers that make it challenging to adhere to SM. SMI with adequate support enhances patients’ self-efficacy.	**Discordance** Indirectly, SM burden can be measured by how patients valued diet and exercise, intensive SM and usual care. No difference was showed between intensive blood glucose control and usual care. A possible explanation is that these values were obtained in clinical trials. In contrast, qualitative findings describe the extra burden patients experience especially when start integrating SM in everyday life.
**Hypoglycaemia**	Hypoglycaemia values varied according to severity. The worst values were for major hypoglycaemia and events with very severe symptoms or presented at night. Mean utility values ranged from 0.540 (95% CI: 0.500 to 0.580) for very severe hypoglycaemic symptoms to 0.800 (95% CI: 0.760 to 0.840) for hypoglycaemic non-severe symptoms. Major hypoglycaemia impacts three times more than minor hypoglycaemia (0.159 (SD 0.11) and − 0.045 (SD 0.028), respectively). In Discrete choice experiment (DCE) studies, willingness to pay to avoid hypoglycaemia varied from 45 US$ to 104 US$/month, with higher values for night-time events (72 US$ to 94 US$) [90, 104, 112] and for one event less of major hypoglycaemic per year (80 US$ to 104 US$) [112].	Many patients do not experience warning signs of hypoglycaemia, and it is not easy for them to understand their new bodily reactions. Hypoglycaemia is a significant concern for patients, impacting their emotional state, daily functioning and engagement with insulin. Insulin-related nocturnal hypoglycaemia is associated with a disrupting effect on diabetes SM, including sleep quality and next-day functioning, work performance and driving, negative financial consequences and quality of life or personal well-being. Hypoglycaemia causes fear and is associated with reduced adherence to treatment and high blood glucose levels. Hypoglycaemia represents a challenge for patients and their families. Family members may need to take control in acute situations, for which they need information and resources [43, 46, 52, 54, 56–59, 78, 84–86, 88].	**Confirmation** Both sources of evidence describe how burdensome hypoglycaemia is, especially nocturnal hypoglycaemia. The impact of severity is better described in quantitative data, whereas qualitative findings confirm the significant concern that hypoglycaemia represents, the implications for quality of life, and adherence to treatment and SM.
***Quality of life*** ***and Psychological distress***		Patients with T2DM usually feel a myriad of emotions related to the diabetes diagnosis, complications and SM, often overwhelmingly negative. They perceive a loss of confidence, the disruption of usual roles, and a sense of independence. Most patients feel that the disease is “taking over their lives”, threatening how they identify themselves or their “sense of identity”. Diabetes stigma, or patients’ expressions of embarrassment and moral failure associated with T2DM, can be related to the diagnosis, complications and medication use. Stigma may prevent patients from disclosing their diagnosis, impairing their ability to self-manage and negatively influencing medicine-taking behaviour, especially insulin treatment. When accepting the disease, patients can feel supported by others. Family is an essential motivator to adhere to diabetes SM. However, for some patients, family support is perceived as interference. Talking to similar others and sharing experiences is an essential source of emotional support [41, 43–49, 51, 53–58, 70, 71, 73, 78, 80, 84–86, 88, 89].	No data was found in quantitative utility-based measures.
***Lipid control***		Some patients do not perceive the benefit of taking medicines for lipid control. The absence of symptoms and the lack of awareness of the increased risk for cardiovascular disease are potential explanations for this attitude [65, 71, 104].	No data was found in quantitative utility-based measures.

*Utilities are measured on a scale from 0 = death to 1 = perfect health. These values can also be expressed as “willingness to pay” or money patients would pay to avoid or get an expected outcome

In weight change, findings also expanded each other. Utility measures referred to a higher importance of extreme obesity than being overweight, while non-utility measures referred to the fear of weight changes concerning treatment consequences. Long-term complications were more detailed by utility-based measures. The most important outcomes for patients were diabetic neuropathic pain, blindness and amputation; in qualitative findings, we also found evidence for diabetic foot ulceration, amputation and cardiovascular risk. The outcome burden of SM included findings from adherence to a healthy diet, physical activity, and treatment, quality of life, scheduled care, and experience of care. Quantitative utility-based findings were discordant. This difference can be explained by the indirectness in the population and measures considered. Hypoglycaemia findings were complementary and confirmatory; on the one hand, a body of evidence expressed the outcomes’ importance in terms of severity, and on the other hand, qualitative evidence informed the experience and the difficulties in detecting, treating and preventing this event. Non-utility measures informed lipid control and quality of life.

## Summary

The identified research evidence shows there is important variability in how patients with T2DM value critical outcomes, mainly influenced by contextual factors and the degree of disease progression. SM is possible when patients can adjust and accept their diagnosis and treatment; furthermore, health literacy is critical since it unlocks healthcare access. Knowledge provision is better received in a positive patient-HCP relationship within a culturally sensitive approach. In this sense, the decision-making process with HCPs enables patients’ engagement in SM. SMIs with adequate support enhance patients’ self-efficacy; however, it requires building capabilities, behavioural skills, social support, and scheduled care.

It is difficult for most patients and their informal caregivers to perceive the risk of long-term complications. Being able to perform self-monitoring facilitates awareness of glycaemic complications and glucose control; however, fear of hypoglycaemia and weight change may hinder adherence to treatment. The loss of quality of life, physical and psychological constraints, associated costs, comorbidities and misleading life expectancy beliefs represent barriers to SMIs. Thus, to integrate SM into everyday life, patients and informal caregivers require tailored, contextualized, self-paced training.

## Discussion

### Main findings

We synthesised 54 SRs of diverse nature on how patients perceive or experience outcomes when dealing with SM, corresponding to 939 studies across 19 countries. We found important variability in how patients with T2DM and their informal caregivers value critical outcomes of SMIs, mainly influenced by contextual factors and disease progression. SM is possible when patients can adjust and accept their diagnosis and treatment. It requires building capabilities, behavioural skills, social support, and attending to scheduled care. Patients and informal caregivers need contextualized self-paced training to integrate SM into everyday life.

The themes frequently overlapped since qualitative research findings are not necessarily neat and discrete. Family informal caregivers can also be affected by the SM process and financial requirements of diabetes care. They are involved when patients use SMIs and recognise having limited knowledge to engage with SM successfully. The family acts as a facilitator of SM, especially when they need to assume responsibility for the patient’s health in acute events. However, in some cases, non-supportive family interactions limit SMIs’ effectiveness, adding extra burden for patients. Most patients from ethnic minorities living in Western countries prefer to receive support that considers their cultural beliefs and traditions. Culturally tailored interventions are generally perceived as facilitators.

When comparing utility and qualitative findings, the former informed the direction of patients’ preferences and the typology and severity of outcomes of SMIs (e.g., severe hypoglycaemia vs. mild one or extreme obesity vs. overweight). The latter provided information regarding the burden associated with SM (e.g., barriers to adhering to a healthy diet, or physical activity).

#### Our findings in the context of previous research

Our results are consistent with findings from previous metareviews, exploring the perspectives of patients with other chronic diseases [19, 92–94]. Overall, patients’ perspectives on SMIs vary according to the disease stage and the specific SM process, which requires time and deliberate effort to integrate into daily life [19]. These perspectives are influenced by contextual factors, including the perceived benefit and usability of the intervention, the sense of community, and the level of individualised care [19, 92]. Patients with hypertension also perceived adherence to SM as challenging [94]. Barriers to SM included familial (lack of support, need for separate meals), environmental (sense of security, local amenities, healthy food availability), financial status and logistical (frequency of appointments, work schedules, accessibility) reasons. The reviews also identified a degree of deliberateness in non-adherent behaviours, which was influenced by the perception of symptoms, the disease severity, stress or fear of dependency. A common finding across reviews, is that having a collaborative, supportive relationship between patients and HCPs is crucial for effective SM [93, 94].

Previous reviews that included HCPs’ perspectives found that their views complement patients’ and informal caregivers’. HCPs in diabetes care considered the main barriers to implementing SM were the limited resources, heavy workloads and environmental constraints [19, 95]. Consultation time was the most frequently reported factor [56, 60, 96, 97]. HCPs often reported being unable to address patients’ broader psychosocial aspects of diabetes care, because of a lack of experience or training in effective communication, counselling, goal setting and shared decision-making [47, 60]. Some described experiencing frustration around patient compliance; and inadequacy and helplessness at being unable to address psychosocial concerns [58, 95], and others considered non-adherence primarily as patients’ own failure [60].

Our findings resonate with theoretical frameworks that explain attitudes and behaviours in patients with chronic conditions. These include the “Common sense model of self-regulation of health and illness” [98], “The shifting perspectives model of chronic illness [99], “the integrative model of behaviour prediction” [100] and the “Health Belief Model” [101]. Consistent with these frameworks and their different but complementary components, our findings described that a crucial step to start with SM is adopting the diabetic identity. The existence of barriers such as limited knowledge, weight change beliefs, the fear of insulin injections, fatalistic beliefs, costs or quality of life constraints can limit the chances of seeking treatment. However, being aware of the importance of outcomes and starting self-monitoring can help to gain insight into their disease, and learn the influence of lifestyle behaviours and treatment. Shifting processes, such as changes in treatment requirements and acute events, can affect adherence to SM. The experience of an acute event or long-term complications could represent cues to action where patients can find SMIs valuable, especially when they start perceiving benefits and self-efficacy.

#### Strengths and Limitations

Our study has several strengths, including using mixed-methods, selecting a broad scope based on the principles of SM, and considering SM as a continuous process transversal to the diabetes journey instead of limiting to the term “self-management” or focusing only on SMIs. We also applied a sensitive search using a validated content search strategy. We also applied rigorous iterative methods to collect and analyse data, and ensure a balanced interpretation. We provided detailed information on the outcomes of T2DM SMI and proposed a logical sequence of findings to explain how outcomes interact and their relevance in the SM process. The high level of data saturation for some outcomes, with only a slight overlap across reviews (CCA = 0.73%), increases the credibility of the findings.

Our study also has some limitations. Since we analysed data based on second-order findings, we acknowledge that individual SRs authors’ analyses could influence our results. However, data saturation for some outcomes makes this potential source of bias unlikely. Moreover, obtaining and reanalysing first-order findings (primary data) was beyond the scope of this overview. Despite most included SRs being high quality and applying rigorous and transparent synthesis methods, the high variability of data reporting has limited us from providing complete information regarding the quality of primary studies and population characteristics. Since we included only English publications, sample origins included patients mostly from high-income countries. Our findings might lack generalisability to patients from low or middle-income countries. Finally, given the inconsistent reporting of information, we could not evaluate the certainty of evidence.

## Implications for practice and research

SMIs are complex and context-specific interventions that require an in-depth understanding of the unique experiences of specific T2DM subpopulations. We propose recommendations for policymakers and HCPs when developing SMIs For example, the need to develop culturally sensitive programmes to facilitate patients’ adaptation to T2DM diagnosis, treatment, and decision-making with HCPs (Table 4). The main areas of improvement include providing psychological support, tailoring messages to health literacy and ethnic considerations, detecting people at high risk early and supporting patients based on their specific needs at different stages.

**Table 4:** Recommendations for Policy, Practice and Future Research

*Barriers/Challenges*	*Recommendations*
Patients require personal adjustments to accept the diagnosis and deal with treatment	• Develop programmes that include psychological interventions for patients with recently diagnosed T2DM. • Provide close support during the early stages of T2DM diagnosis.
Patients with low numeracy or literacy skills can find it difficult accessing to healthcare services	• Include patients in the research and development of educational programmes or materials to test their comprehension and usability. • Facilitate materials considering numeracy and literacy differences.
Patients value highly educational interventions that answer their information needs and have considered their training preferences and beliefs on health	• Incorporate a culturally sensitive approach in SMI development. • Reinforce strategies for positive patient-HCP relationships and understanding what matters most to patients. • Provide tailored messages according to patients’ characteristics and context.
Engaging in SMIS is possible with shared decision-making	• Reinforce programs for effective communication. • Develop a culturally sensitive mindset in the healthcare organization. • Include patients in their care decisions to find a common agenda. • Avoid time pressures as much as possible Try different modalities for scheduling visits according to patients’ requirements, e.g., more frequent visits.
Medication adherence is perceived as requiring self-regulation and deliberate effort. Fear is always present in treatment adherence. Fear of hypoglycaemia and weight change may hinder treatment adherence.	• Offer alternatives that allow patients to adapt treatment to their activities, preferring simplified regimens. • Offer a self-paced process of incorporation of treatment. • Support patients with difficulties, solve doubts about treatment effectiveness, and how to face eventual complications.
Patients do not readily perceive the risk of long-term complications.	• Educate patients about the silent progress of diabetes when it is not controlled.
Quality of life constraints and physical and psychological barriers can make it challenging to follow SM. In some cases, they perceive or experience diabetes stigma.	• Identify early patients at higher risk of not getting support to face diabetes psychological and emotional burden. • Facilitate or develop programs to help patients deal with the changes in adapting to diabetes.
Most patients find it more feasible to adhere to a healthy diet when they have practical Knowledge regarding diet and cooking, develop self-discipline, become more proactive, and have social support.	• Provide practical tips for diet and cooking. • Inquire about their level of social support when dealing with adherence to a healthy diet.
Patients find it easier to engage and continue with physical activity when they perceive having social support, having experienced benefits from exercise or having reasonable expectations, and gaining self-efficacy.	• Help to set reasonable goals with physical activity programs. • Inquire about their level of social support to start and continue with physical activity.
Talking to peers and sharing experiences is an essential source of emotional support and facilitates SM integration in their daily life.	• Recommend participating in peer-support groups for patients with diabetes and related complications.

We identified some quantitative and qualitative research gaps, reflecting either the lack of SRs or primary studies in these areas. The underresearched outcome in both types of evidence was unscheduled care. In the qualitative branch, we found scarce evidence for comorbidities, hyperglycaemia, lipid control, and complications such as cardiovascular diseases, neuropathic pain, renal disease, and stroke. On the other hand, we consider some themes close to saturation, such as barriers to SM, adherence to treatment/insulin, and SM in ethnic minorities. In the quantitative utility-based research, we did not find evidence for psychological distress and lipid control, and scarce findings for the burden of SM and the importance of glycemic control.

Summaries can vary in extension or detail according to the outcome definition or the scope. Our findings can inform the formulation of recommendations for other healthcare decisions in diabetes care. These findings can also inform the development of educational materials and decision-making tools.

## Conclusion

Our results represent what patients with T2DM and their informal caregivers perceive as most important when dealing with SMIs. Their perspectives on SMI outcomes are variable since they are affected by the progression of the disease and several contextual factors. We found quantitative or qualitative evidence for almost all outcomes of the core outcome set of T2DM SMIs. Considering the availability of different types of SMIs, decision-making must incorporate patients’ and informal caregivers’ values and preferences on the importance of SMIs outcomes. We have summarised available SRs on this topic and identified some research gaps. Our results can facilitate the development and evaluation of SMIs, and guide decision-making in diabetes care, including the formulation of decisions and recommendations.

### Electronic supplementary material

Below is the link to the electronic supplementary material.

Additional file 1: Definitions of outcomes for Self-Management interventions

Additional file 2: Search strategy

Additional file 3: Reasons for the exclusion of references reviewed in full text

Additional file 4: General characteristics of included SRs

Additional file 5: JBI Critical Appraisal Checklist assessment

Additional file 6: Overlap analysis

## Data Availability

The datasets supporting the conclusions of this article are included within the article, its additional files, and the online dataset Mixed-methods overview T2DM. See: https://osf.io/dj3wy/?view_only=f0f3a82ea97747b59beb4de2a11c05f8.

## Abbreviations

CCA: Corrected covered area
JBI: Joanna Briggs Institute
HCPs: Healthcare providers
SM: Self-management
SMBG: Self-monitoring of blood glucose
SMIs: Self-management interventions
SR: Systematic review
T1DM: Type 1 Diabetes Mellitus
T2DM: Type 2 Diabetes Mellitus
