# Supplementary material for: Patients’ and informal caregivers’ perspectives on self-management interventions for type 2 diabetes mellitus outcomes: a mixed-methods overview of 14 years of reviews
Source: Arch Public Health. 2023 Aug 4;81:140. doi: 10.1186/s13690-023-01153-9 (PMC10401891; doi:10.1186/s13690-023-01153-9)
Supplement: Supplementary file 1 — Additional file 1: Definitions of outcomes for Self-Management interventions [file 13690_2023_1153_MOESM1_ESM.pdf]

Additional file 1

Patients' and informal caregivers' perspectives on Self-Management Interventions for Type 2 Diabetes Mellitus outcomes: a mixed-methods overview of 14 years of reviews

Definitions of Outcomes for Self-Management interventions

The following outcomes were selected as the Core Outcome Set (COS) for SMI in T2DM. The selection process included two rounds of Delphi survey with patients, and a consensus meeting included research experts, clinicians, policymakers and patients' representatives.

For our analysis, we incorporate psychological functioning into quality of life and personalised care into overall satisfaction with SMI (renamed as the experience of care)

n	COS outcomes T2DM	Definition
**1**	Knowledge	Familiarity with or awareness or understanding of facts, information, and descriptions and skills acquired through experience or education involving perceiving, discovering, and learning
**2**	Health literacy	Having the cognitive and social skills that determine a person's ability to find health-related information, understand the information, judge its trustworthiness, and to take appropriate action in everyday life (for example regarding lifestyle choices, self-care and so on).
**3**	Self-efficacy	A person's belief that s/he can do something, often related to a specific goal s/he wants to achieve, feeling of confidence and being in control
**4**	Patient activation	It is the degree a patient understands their role in the healthcare process and their level of knowledge, skill, and confidence in managing their health. Patient activation involves four stages: (1) believing the patient has an important role, (2) having the confidence and knowledge necessary to take action, (3) taking action to maintain and improve one's health, and (4) staying the course even under stress.
**5**	Dietary habits	Healthy eating habits and the extent to which a person adheres to dietary recommendations agreed on with a healthcare provider
**6**	Physical activity	Any activity that gets a patient up and about. It includes everyday activities like walking to the shops, gardening or housework, and leisure activities such as riding a bike, swimming or playing football. This element also covers the extent to which a person adheres to a physical activity plan agreed on with a healthcare provider.
**7**	Adherence (to treatment)	Includes medication use, which refers to the degree to which a patient takes their medication properly (administration, timing, etc.), and medication adherence, which refers to the degree to which a patient adheres to medication use recommendations agreed on with a healthcare provider. For correct medication use and adherence, patients/caregivers

		need to know how and when a medication should be taken, what should be avoided, and what negative effects should be looked out for.
**8**	Self-monitoring	Tracking one's symptoms and or behaviours. In some cases, this may involve subsequent adjustment of behaviours.<br>Examples: Monitoring of blood sugar levels, peak flow recording, tracking of calorie intake.<br>Outcome measures for assessing patient or carer adherence to expected clinical self-management behaviours
**9**	(other) Self-management behaviours	Disease-specific behaviours: Activities aimed at achieving better management of a specific condition.<br>Example: Foot care for patients with diabetes. Early recognition of symptoms Understanding of and appropriate and timely monitoring of symptoms.<br>Example: A list of worrisome symptoms, including indications of when it is necessary to contact the doctor's office or go to the emergency department.<br>Asking for professional help or emergency care when needed The ability of patients or caregivers to readily ask for professional help or emergency care, to accept that they sometimes need help, and to clearly communicate this message. Learning to ask for help without feeling weak, incompetent, or like a burden. Asking for help and doing so in a timely manner will enhance patient wellbeing.<br>Device Management Appropriate use of self-management devices, including knowing how to use a device and manage tasks and knowing when and how to ask for technical support or help.<br>Examples: Use of an insulin pump, telehealth care system.
**10**	HbA1C	Higher amounts of glycated haemoglobin indicates poorer control of blood glucose levels
**11**	Weight (management)	Weight (management) 1) Weight loss: Reduction in weight. 2) Stable weight: Being able to keep a stable weight. 3) Waist size: Measure of waist circumference
**12**	Blood pressure (Co-morbidities)	Systolic pressure<br>Diastolic pressure
**13**	Lipid Profile	Fasting Cholesterol (mmol/L)<br>LDL<br>HDL<br>triglycerides<br>Total Cholesterol<br>Total cholesterol: HDL ratio
**14**	Long-term complications	Unfavourable progression or consequence of a long-term condition. The long-term effects of diabetes mellitus include the progressive development of the specific complications of retinopathy with potential blindness, nephropathy that may lead to renal failure, and/or neuropathy with risk of foot ulcers, amputation, Charcot joints, and features of autonomic dysfunction, including sexual dysfunction. People with diabetes are at increased risk of cardiovascular, peripheral vascular, and cerebrovascular disease
**15**	Hyperglycemia	Hyperglycemia
**16**	Hypoglycaemia	Hypoglycaemia event

**17**	Life expectancy	Mortality outcome measures include the number of deaths by place, time, or cause observed before, during, or after the implementation of a self-management intervention.
**18**	Overall quality of life	An individual's perception of their position in life in the context of the culture and value systems in which they live and in relation to their goals, expectations, standards and concerns. Quality of life (with a special focus on being able to perform usual activities, the burden of treatment, mobility, pain or discomfort, coping, happiness and self-esteem). Including but not limited to the diabetes-specific quality of life measures
**18.1**	Psychological functioning	Measures of anxiety, depression, etc. Also includes the extent to which physical health or emotional problems interfere with normal social activities with family, friends, neighbours, or groups.
**19**	Experience of care: Overall satisfaction with self-management interventions	General feeling of satisfaction that patients and caregivers have with a self-management intervention in which they are participating
**19.1**	Experience of care: Personalised care	The extent to which a patient's needs and preferences are taken into account.
**20**	Participation and decisions making	Feeling able to participate actively in her/his own care and treatment decisions (as much as s/he wishes).
**21**	Scheduled care	Visits or contact with healthcare providers, including primary care, outpatient visits, etc. In this outcome, we considered any findings related to the number of visits to GPs or Nurses. If the included articles specified this, we made this distinction. However, we also considered results related to non-specified healthcare providers or professionals
**22**	Unscheduled care	Number of unplanned or emergency visits/admissions. Hospitalisation and rehospitalisation (usually defined as a new admission within 30 days of discharge).
**23**	Value for money of the self-management intervention (healthcare returns)	Costs to the healthcare system or savings achieved as a result of a self-management intervention.
