# Supplementary material for: Patients’ and informal caregivers’ perspectives on self-management interventions for type 2 diabetes mellitus outcomes: a mixed-methods overview of 14 years of reviews
Source: Arch Public Health. 2023 Aug 4;81:140. doi: 10.1186/s13690-023-01153-9 (PMC10401891; doi:10.1186/s13690-023-01153-9)
Supplement: Supplementary file 2 — Additional file 2: Search strategy [file 13690_2023_1153_MOESM2_ESM.pdf]

*Additional file 2*

# Patients' and informal caregivers' perspectives on Self-Management Interventions for Type 2 Diabetes Mellitus outcomes: a mixed-methods overview of 14 years of reviews

***Search strategy***

We included terms designed explicitly for values and preferences studies [1] combined with MeSH terms and free text for Type 2 Diabetes Mellitus. We adapted the MEDLINE strategy for each database using database-specific headings. We applied the review filters available in each database.In Medline, the filter represents a search strategy intended to retrieve citations to systematic reviews in PubMed and encompasses: citations assigned the "Systematic Review" publication type during MEDLINE indexing; citations that have not yet completed MEDLINE indexing; and non-MEDLINE citations [2].

**Table S1. Search terms**

| **MEDLINE (PubMed)** |
|---|
| #1 perceive*[tiab] |
| #2 perception*[ti] |
| #3 experience*[ti] |
| #4 perspective*[tiab] |
| #5 understand*[tiab] |
| #6 preference*[tiab] |
| #7 value*[tiab] |
| #8 choice*[tiab] |
| #9 (#1 OR #2 OR #3 OR #4 OR #5 OR #6 OR #7 OR #8) |
| #10 discrete choice*[tiab] |
| #11 decision*[tiab] |
| #12 Decision Making[MAJR] |
| #13 (#10 OR #11 OR #12) |
| #14 (health[ti] AND utilit*[ti]) |
| #15 “Decision Support Techniques”[MeSH] |
| #16 gamble*[tiab] |
| #17 prospect theory[tiab] |
| #18 preference score[tiab] |
| #19 preference elicitation[tiab] |
| #20 health utilit*[tiab] |
| #21 utility value*[tiab] |
| #22 Utility score*[tiab] |
| #23 Utility estimate*[tiab] |
| #24 health state utilit*[tiab] |
| #25 health state[tiab] |
| #26 feeling thermometer*[tiab] |

| |
|---|
| #27 best worst scaling[tiab] |
| #28 standard gamble[tiab] |
| #29 time trade-off[tiab] |
| #30 TTO[tiab] |
| #31 probability trade-off[tiab] |
| #32 “Patient Preference”[MeSH] |
| #33 (#14 OR #15 OR #16 OR #17 OR #18 OR #19 OR #20 OR #21 OR #22 OR #23 OR #24 OR #25 OR #26 OR #27 OR #28 OR #29 OR #30 OR#31 OR #32) |
| #34 preference based [tiab] |
| #35 preference score*[tiab] |
| #36 multiattribute[tiab] |
| #37 multi attribute[tiab] |
| #38 EuroQol 5D[tiab] |
| #39 EuroQol5D[tiab] |
| #40 EQ5D[tiab] |
| #41 EQ 5D[tiab] |
| #42 SF6D[tiab] |
| #43 SF 6D[tiab] |
| #44 HUI[tiab] |
| #45 15D[tiab] |
| #46 (#34 OR #35 OR #36 OR #37 OR #38 OR #39 OR #40 OR #41 OR #42 OR #43 OR #44 OR #45) |
| #47 (#13 OR #33 OR #46) |
| #48 meta-ethnograph*[tiab] |
| #49 metasynthes*[tiab] |
| #50 meta-synth*[tiab] |
| #51 (synthes*[ti] AND qualitative[ti]) |
| #52 (review[ti] AND qualitativ*[ti]) |
| #53 (#48 OR #49 OR #50 OR #51 OR #52) |
| #54 systematic[sb] |
| #55 (#53 OR #54) |
| #56 (#9 AND #55) |
| #57 (#47 AND #54) |
| #58 (#56 OR #57) |
| #59 "Diabetes Mellitus"[Mesh] |
| #60 diabet*[tiab] |
| #61 (#59 OR #60) |
| #62 (#58 AND #61) |
| **CINAHL** |
| #1 TI meta-ethnograph* OR AB meta-ethnograph* |
| #2 TI metasynthes* OR AB metasynthes* |
| #3 TI meta-synth* OR AB meta-synth* |
| #4 TI "meta aggregat*" OR AB "meta aggregat*" |
| #5 TI "thematic synthes*" OR AB "thematic synthes*" |
| #6 TI synthes* qualitative |
| #7 TI review AND TI qualitative |

| |
|---|
| #8 (#1 OR #2 OR #3 OR #4 OR #5 OR #6 OR #7) |
| #9 TI perceive* OR AB perceive* |
| #10 TI perception* OR AB perception* |
| #11 TI expererience* OR AB experience* |
| #12 TI perspective* OR AB perspective* |
| #13 TI understand* OR AB understand* |
| #14 TI preference* OR AB preference* |
| #15 (#9 OR #10 OR #11 OR #12 OR #13 OR #14) |
| #16 (#15) Limiters – Clinical queries: Review - Best Balance |
| #17 (#8 AND #15) |
| #18 TI value* OR AB value* |
| #19 TI choice* OR AB choice* |
| #20 TI "patient* view*" OR AB "patient* view*" |
| #21 TI decision* OR AB decision* |
| #22 TI "health utilit*" OR AB "health utilit*" |
| #23 MH "Attitude to Health+" |
| #24 MH "Patient Preference+ |
| #25 MH "Decision Making+ |
| #26 (#18 OR #19 OR #20 OR #21 OR #22 OR #23 OR #24 OR #25) |
| Limiters – Clinical queries: Review - Best Balance |
| #27 (#16 OR #17 OR #26) |
| #28 MH Diabetes Mellitus+ |
| #29 TI diabet* |
| #30 (#28 OR #29) |
| #31 (#30 AND #27) Limiters - Exclude MEDLINE registries |
| **PsycINFO** |
| #1 TI meta-ethnograph* OR AB meta-ethnograph* |
| #2 TI metasynthes* OR AB metasynthes* |
| #3 TI meta-synth* OR AB meta-synth* |
| #4 TI meta aggregat* OR AB meta aggregat* |
| #5 TI thematic synthes* OR AB thematic synthes* |
| #6 TI synthes* AND TI qualitative |
| #7 TI review AND TI qualitative |
| #8 (#1 OR #2 OR #3 OR #4 OR #5 OR #6 OR #7) |
| #9 TI perceive* OR AB perceive* |
| #10 TI perception* OR AB perception* |
| #11 TI perspective* OR AB perspective* |
| #12 TI understand* OR AB understand* |
| #13 TI preference* OR AB preference* |
| #14 (# 9 OR #10 OR #11 OR #12 OR #13) |
| #15 (#14 AND #8) |
| #16 (#14) Limiters Methodology Literature review |
| #17 TI value* OR AB value* |
| #18 TI choice* OR AB choice* |
| #19 TI patient* view* OR AB patient* view* |

| |
|---|
| #20 TI decision* OR AB decision* |
| #21 TI "health utilit*" OR AB "health utilit*" |
| #22 DE "Health Attitudes" |
| #23 DE "Preferences" OR DE "Preference Measures" |
| #24 DE "Decision Making" |
| #25 (#17 OR #18 OR #19 OR #20 OR #21 OR #22 OR #23 OR #24) |
| #26 (#25) Limiters Methodology Literature review |
| #27 (#15 OR #16 OR #26) |
| #28 MA diabetes mellitus |
| #29 TI diabet* |
| #30 MM "Diabetes Mellitus" |
| #31 (#28 OR #29 OR #30) |
| #32 (#31 AND #27) |

***References***


1. Selva A, Sola I, Zhang Y, Pardo-Hernandez H, Haynes RB, Martinez Garcia L et al. Development and use of a content search strategy for retrieving studies on patients' views and preferences. Health Qual Life Outcomes. 2017;15(1):126. doi:10.1186/s12955-017-0698-5.

2. Search Strategy Used to Create the PubMed Systematic Reviews Filter. https://www.nlm.nih.gov/bsd/pubmed_subsets/sysreviews_strategy.html
