# Supplementary material for: Patients’ and informal caregivers’ perspectives on self-management interventions for type 2 diabetes mellitus outcomes: a mixed-methods overview of 14 years of reviews
Source: Arch Public Health. 2023 Aug 4;81:140. doi: 10.1186/s13690-023-01153-9 (PMC10401891; doi:10.1186/s13690-023-01153-9)
Supplement: Supplementary file 3 — Additional file 3: Reasons for the exclusion of references reviewed in full text [file 13690_2023_1153_MOESM3_ESM.pdf]

Additional file 3

Patients' and informal caregivers' perspectives on Self-Management Interventions for Type 2 Diabetes Mellitus outcomes: a mixed-methods overview of 14 years of reviews

Reasons for the exclusion of references reviewed in full text

	Author	Reason
1.	Alleman CJ, Westerhout KY, Hensen M, Chambers C, Stoker M, Long S, et al. Humanistic and economic burden of painful diabetic peripheral neuropathy in Europe: A review of the literature. Diabetes Res Clin Pract. 2015;109(2):215-25. doi: 10.1016/j.diabres.2015.04.031.	Different method of evaluation
2.	Alsairafi ZK, Taylor KM, Smith FJ, Alattar AT. Patients' management of type 2 diabetes in Middle Eastern countries: review of studies. Patient preference and adherence. 2016;10:1051-62.	Different study design
3.	Bauer, J., et al. (2015). "Impact of mHealth chronic disease management on treatment adherence and patient outcomes: a systematic review." JMIR Mhealth Uhealth 17(2): e52.	Different population
4.	Beaudet A, Clegg J, Thuresson PO, Lloyd A, McEwan P. Review of utility values for economic modeling in type 2 diabetes. Value Health. 2014;17(4):462-70. doi: 10.1016/j.jval.2014.03.003.	Different method of evaluation
5.	Brennan VK, Mauskopf J, Colosia AD, Copley-Merriman C, Hass B, Palencia R. Utility estimates for patients with Type 2 diabetes mellitus after experiencing a myocardial infarction or stroke: a systematic review. Expert Rev Pharmacoecon Outcomes Res. 2015;15(1):111-23. doi: 10.1586/14737167.2015.965152.	Different method of evaluation
6.	Brewster S, Bartholomew J, Holt RIG, Price H. Non-attendance at diabetes outpatient appointments: a systematic review. Diabetic Medicine. 2020;37(9):1427-42.	Different phenomenon of interest
7.	Brewster S, Bartholomew J, Holt RIG, Price H. Non-attendance at diabetes outpatient appointments: a systematic review. Diabetic Medicine. 2020;37(9):1427-42.	Different phenomenon of interest
8.	Brindle T, Farmer P. Undisturbed wound healing: a narrative review of the literature and clinical considerations. Wounds International. 2019;10(2):40-8.	Different study design
9.	Brundisini, F., et al. (2015). "Type 2 diabetes patients' and providers' differing perspectives on medication nonadherence: a qualitative meta-synthesis." BMC Health Serv Res 15: 516.	Different phenomenon of interest
10.	Bullen B, Young M, McArdle C, Ellis M. Overcoming barriers to self-management: The person-centred diabetes foot behavioural agreement. Foot. 2019;38:65-9.	Different phenomenon of interest

11. Bunn, F., et al. (2017). "What works for whom in the management of diabetes in people living with dementia: a realist review." BMC Med 15(1): 141.	Different population
12. Byrne, M., et al. (2017). "A core outcomes set for clinical trials of interventions for young adults with type 1 diabetes: an international, multi-perspective Delphi consensus study." Trials 18(1): 602.	Different population
13. Carter DD, Robinson K, Forbes J, Hayes S. Experiences of mobile health in promoting physical activity: A qualitative systematic review and meta-ethnography. PLoS One. 2018;13(12):e0208759.	Different study design
14. Castonguay A, re, Miquelon P, Boudreau F. Self-regulation resources and physical activity participation among adults with type 2 diabetes. Health Psychology Open. 2018;5(1).	Different study design
15. Cheng LJ, Wang W, Lim ST, Wu VX. Factors associated with glycaemic control in patients with diabetes mellitus: A systematic literature review. Journal of Clinical Nursing (John Wiley & Sons, Inc). 2019;28(9):1433-50.	Different study design
16. Chima CC, Abdelaziz A, Asuzu C, Beech BM. Impact of Health Literacy on Medication Engagement Among Adults With Diabetes in the United States: A Systematic Review. Diabetes Educator. 2020;46(4):335-49.	Different phenomenon of interest
17. Choi YJ, Smaldone AM. Factors Associated With Medication Engagement Among Older Adults With Diabetes: Systematic Review and Meta-Analysis. Diabetes Educator. 2018;44(1):15-30.	Different study design
18. de Andrade Hovadick AC, Reis IA, Carvalho Torres H. Short Message Service (SMS) and self-care promotion in type 2 DM: an integrative review. Acta Paulista de Enfermagem. 2019;32(2):210-9.	Different phenomenon of interest
19. Flôr CR, Baldoni NR, Aquino JA, Baldoni AO, Fabbro ALD, Figueiredo RC, et al. What is the association between social capital and diabetes mellitus? A systematic review. Diabetes Metab Syndr.12(4):601-5.	Different phenomenon of interest
20. Frier A, Devine S, Barnett F, Dunning T. Utilising clinical settings to identify and respond to the social determinants of health of individuals with type 2 diabetes—A review of the literature. Health & Social Care in the Community. 2020;28(4):1119-33.	Different study design
21. Ganasegeran K, Hor CP, Jamil MFA, Loh HC, Noor JM, Hamid NA, et al. A Systematic Review of the Economic Burden of Type 2 Diabetes in Malaysia. Int J Environ Res Public Health.17(16).	Different study design
22. Gask, L., et al. (2011). "What is the relationship between diabetes and depression? a qualitative meta-synthesis of patient experience of co-morbidity." Chronic Illn 7(3): 239-252.	Different population
23. Gillett, M., et al. (2010). "Delivering the diabetes education and self management for ongoing and newly diagnosed (DESMOND) programme for people with newly diagnosed type 2 diabetes: cost effectiveness analysis." Bmj 341: c4093.	Different study design
24. Golden, S. H., et al. (2017). "The Case for Diabet es Population Health Improvement: Evidence-Based Programming for Population Outcomes in Diabetes." Curr Diab Rep 17(7): 51.	Different study design

25. Greenwood, D. A., et al. (2014). "Telehealth Remote Monitoring Systematic Review: Structured Self-monitoring of Blood Glucose and Impact on A1C." J Diabetes Sci Technol 8(2): 378-389	Different phenomenon of interest
26. Gucciardi, E. (2008). "A systematic review of attrition from diabetes education services: strategies to improve attrition and retention research." Canadian Journal of Diabetes 32(1): 53-65.	Different phenomenon of interest
27. Hamine, S., et al. (2015). "Impact of mHealth chronic disease management on treatment adherence and patient outcomes: A systematic review." Journal of Medical Internet Research 17(2)	Different population
28. Hennink, M. M., et al. (2017). "How are qualitative methods used in diabetes research? A 30-year systematic review." Glob Public Health 12(2): 200-219.	Different phenomenon of interest
29. Hogg, F. R., et al. (2012). "Measures of health-related quality of life in diabetes-related foot disease: a systematic review." Diabetologia 55(3): 552-565.	Different phenomenon of interest
30. Jaam M, Awaisu A, Mohamed Ibrahim MI, Kheir N. A holistic conceptual framework model to describe medication adherence in and guide interventions in diabetes mellitus. Research in Social & Administrative Pharmacy. 2018;14(4):391-7.	Different study design
31. Janssen MF, Lubetkin EI, Sekhobo JP, Pickard AS. The use of the EQ-5D preference-based health status measure in adults with Type 2 diabetes mellitus. Diabet Med. 2011;28(4):395-413. doi: 10.1111/j.1464-5491.2010.03136.x.	Different method of evaluation
32. Joy, S. M., et al. (2013). "Patient preferences for the treatment of type 2 diabetes: a scoping review." PharmacoEconomics 31(10): 877-892.	Different phenomenon of interest
33. Kennedy-Martin T, Paczkowski R, Rayner S. Utility values in diabetic kidney disease: a literature review. Curr Med Res Opin. 2015;31(7):1271-82. doi: 10.1185/03007995.2015.1041895.	Different method of evaluation
34. Klein, H. A. and K. D. Lippa (2012). "Assuming control after system failure: Type II diabetes self-management." Cognition, Technology & Work 14(3): 243-251.	Different study design
35. Klösch M, Klösch C, Kundt FS, van der Zee-Neuen A, Dieplinger AM. eHealth systems for the optimised care of patients with type 2 diabetes. British Journal of Nursing. 2020;29(5):274-8.	Different phenomenon of interest
36. Lakerveld J, Palmeira AL, Duinkerken E, Whitelock V, Peyrot M, Nouwen A. Motivation: key to a healthy lifestyle in people with diabetes? Current and emerging knowledge and applications. Diabetic Medicine. 2020;37(3):464-72.	Different phenomenon of interest
37. Lasalvia, P., et al. (2016). "Pen Devices for Insulin Self-Administration Compared With Needle and Vial: Systematic Review of the Literature and Meta-Analysis." J Diabetes Sci Technol 10(4): 959-966.	Different phenomenon of interest
38. Lenz, M., et al. (2007). "Meta-analysis does not allow appraisal of complex interventions in diabetes and hypertension self-management: a methodological review." Diabetologia 50(7): 1375-1383.	Different study design
39. Lian, J. X., et al. (2017). "Systematic review on the cost-effectiveness of self-management education programme for type 2 diabetes mellitus." Diabetes Research & Clinical Practice 127: 21-34.	Different phenomenon of interest

40. Lopez-Class, M. and J. Jurkowski (2010). "The limits of self-management: Community and health care system barriers among Latinos with diabetes." Journal of Human Behavior in the Social Environment 20(6): 808-826.	Different study design
41. Lung TWC, Hayes AJ, Hayen A, Farmer A, Clarke PM. A meta-analysis of health state valuations for people with diabetes: explaining the variation across methods and implications for economic evaluation. Quality of Life Research. 2011;20(10):1669-78. doi: 10.1007/s11136-011-9902-y.	Different method of evaluation
42. McCoy MA, Theeke LA. A systematic review of the relationships among psychosocial factors and coping in adults with type 2 diabetes mellitus. Int J Nurs Sci.6(4):468-77.	Different phenomenon of interest
43. McSharry J, Byrne M, Casey B, Dinneen SF, Fredrix M, Hynes L, et al. Behaviour change in diabetes: behavioural science advancements to support the use of theory. Diabetic Medicine. 2020;37(3):455-63.	Different phenomenon of interest
44. McSharry, J., et al. (2016). "Perceptions and experiences of taking oral medications for the treatment of Type 2 diabetes mellitus: a systematic review and meta-synthesis of qualitative studies." Diabet Med 33(10): 1330-1338.	Different phenomenon of interest
45. Medical Advisory S. Behavioural interventions for type 2 diabetes: an evidence-based analysis. Ont Health Technol Assess Ser. 2009;9(21):1-45.	Different phenomenon of interest
46. Molife, C., et al. (2009). "Assessment of patient-reported outcomes of insulin pen devices versus conventional vial and syringe." Diabetes Technol Ther 11(8): 529-538.	Different method of evaluation
47. Morgan TL, Semenchuk BN, Ceccarelli L, Kullman SM, Neilson CJ, Kehler DS, et al. Self-Compassion, Adaptive Reactions and Health Behaviours Among Adults With Prediabetes and Type 1, Type 2 and Gestational Diabetes: A Scoping Review. Canadian Journal of Diabetes. 2020;44(6):555-.	Different study design
48. Mühlbacher, A. C. and A. Kaczynski (2014). "Patient Preferences in Treatment of Diabetes Mellitus: A Systematic Review of Stated Preference Surveys." Value in Health 17(7): A356-A356.	Different method of evaluation
49. Mushcab, H., et al. (2015). "Web-Based Remote Monitoring Systems for Self-Managing Type 2 Diabetes: A Systematic Review." Diabetes Technol Ther 17(7): 498-509.	Different phenomenon of interest
50. Olsson M, Järbrink K, Divakar U, Bajpai R, Upton Z, Schmidtchen A, et al. The humanistic and economic burden of chronic wounds: A systematic review. Wound Repair Regen.27(1):114-25.	Different phenomenon of interest
51. Oni D. Foot Self-Care Experiences Among Patients With Diabetes: A Systematic Review of Qualitative Studies. Wound Manag Prev.66(4):16-25.	Different study design
52. Paduch, A., et al. (2017). "Psychosocial barriers to healthcare use among individuals with diabetes mellitus: A systematic review." Primary Care Diabetes 11(6): 495-514.	Different phenomenon of interest
53. Palmer, A. J., et al. (2006). "Cost-utility analysis in a UK setting of self-monitoring of blood glucose in patients with type 2 diabetes." Current Medical Research & Opinion 22(5): 861-872.	Different phenomenon of interest
54. Phillips A. Recognising the importance of self-management for people with diabetes. British Journal of Healthcare Management. 2019;25(7):224-9.	Different study design

55. Poku E, Brazier J, Carlton J, Ferreira A. Health state utilities in patients with diabetic retinopathy, diabetic macular oedema and age-related macular degeneration: a systematic review. BMC Ophthalmol. 2013;13:74. doi: 10.1186/1471-2415-13-74.	Different method of evaluation
56. Reaney, M., et al. (2016). "Patient Reported Outcomes (PROs) used in recent Phase 3 trials for Type 2 Diabetes: A review of concepts assessed by these PROs and factors to consider when choosing a PRO for future trials." Diabetes Res Clin Pract 116: 54-67.	Different phenomenon of interest
57. Rees, S. and A. Williams (2009). "Promoting and supporting self-management for adults living in the community with physical chronic illness: A systematic review of the effectiveness and meaningfulness of the patient-practitioner encounter." JBI Libr Syst Rev 7(13): 492-582.	Different population
58. Reynolds, R., et al. (2018). "A systematic review of chronic disease management interventions in primary care." 19(1): 11.	Different phenomenon of interest
59. Ridyard, C. H., et al. (2016). "A Systematic Review of Patients' Perspectives on the Subcutaneous Route of Medication Administration." Patient 9(4): 281-292.	Different phenomenon of interest
60. Roberts, S., et al. (2018). "Economic evaluation of type 2 diabetes prevention programmes: Markov model of low- and high-intensity lifestyle programmes and metformin in participants with different categories of intermediate hyperglycaemia." BMC Medicine 16: 1-12.	Different phenomenon of interest
61. Rose MA, Vukicevic M, Koklanis K, Rees G, hu S, Itsiopoulos C. Experiences and perceptions of patients undergoing treatment and quality of life impact of diabetic macular edema: a systematic review. Psychol Health Med.24(4):383-401.	Different phenomenon of interest
62. Roussos S, Mpompota K. Anxiety In Diabetic Foot Ulcer Patients. Perioperative Nursing. 2020;9(2):61-8.	Different phenomenon of interest
63. Saheb Kashaf, M., et al. (2017). "Shared decision-making and outcomes in type 2 diabetes: A systematic review and meta-analysis." Patient Educ Couns 100(12): 2159-2171.	Different phenomenon of interest
64. Saunders T. Type 2 Diabetes Self-Management Barriers in Older Adults: An Integrative Review of the Qualitative Literature. J Gerontol Nurs.45(3):43-54.	Different phenomenon of interest
65. Schabert, J., et al. (2013). "Social stigma in diabetes: A framework to understand a growing problem for an increasing epidemic." The Patient: Patient-Centered Outcomes Research 6(1): 1-10.	Different study design
66. Seo K, Song Y. Self-stigma among Korean patients with diabetes: A concept analysis. Journal of Clinical Nursing (John Wiley & Sons, Inc). 2019;28(9):1794-807.	Different study design
67. Seuring, T., et al. (2015). "The Economic Costs of Type 2 Diabetes: A Global Systematic Review." PharmacoEconomics 33(8): 811-831.	Different phenomenon of interest
68. Shahin W, Kennedy GA, Stupans I. The impact of personal and cultural beliefs on medication adherence of patients with chronic illnesses: a systematic review. Patient Prefer Adherence. 2019;13:1019-35.	Different population
69. Sherifali, D. and G. Meneilly (2016). "Diabetes Management and Education in Older Adults: The Development of a National Consensus of Key Research Priorities." Can J Diabetes 40(1): 31-34.	Different study design

70. Simmons, L. A., et al. (2014). "Patient engagement as a risk factor in personalised health care: a systematic review of the literature on chronic disease." Genome Med 6(2): 16.	Different phenomenon of interest
71. Skinner TC, Joensen L, Parkin T. Twenty-five years of diabetes distress research. Diabetic Medicine. 2020;37(3):393-400.	Different phenomenon of interest
72. Skinner TC, Joensen L, Parkin T. Twenty-five years of diabetes distress research. Diabetic Medicine. 2020;37(3):393-400.	Different study design
73. Stacey, D., et al. (2017). "Decision aids for people facing health treatment or screening decisions." Cochrane Database Syst Rev 4: Cd001431.	Different phenomenon of interest
74. Stewart, K. D., et al. (2016). "Preference for pharmaceutical formulation and treatment process attributes." Patient Prefer Adherence 10: 1385-1399.	Different phenomenon of interest
75. Swanson V, Maltinsky W. Motivational and behaviour change approaches for improving diabetes management. Practical Diabetes. 2019;36(4):121-5.	Different phenomenon of interest
76. Swanson V, Maltinsky W. Motivational and behaviour change approaches for improving diabetes management. Practical Diabetes. 2019;36(4):121-5.	Different study design
77. Tan Shu-Xian, P., et al. (2010). "The Experience of Hypoglycaemia and Strategies Used For its Management by Community-Dwelling Adults with Diabetes Mellitus: A Systematic Review." JBI Library of Systematic Reviews 8: 1-21.	Different study design
78. Toroski M, Kebriaeezadeh A, Esteghamati A, Karyani AK, Abbasian H, Nikfar S. Patient and physician preferences for type 2 diabetes medications: a systematic review. J Diabetes Metab Disord. 2019;18(2):643-56. doi: 10.1007/s40200-019-00449-4.	Different method of evaluation
79. Toroski M, Kebriaeezadeh A, Esteghamati A, Karyani AK, Abbasian H, Nikfar S. Patient and physician preferences for type 2 diabetes medications: a systematic review. J Diabetes Metab Disord.18(2):643-56.	Different method of evaluation
80. Valencia WM, Florez HJ, Palacio AM. Suitable Use of Injectable Agents to Overcome Hypoglycemia Risk, Barriers, and Clinical Inertia in Community-Dwelling Older Adults with Type 2 Diabetes Mellitus. Drugs & Aging. 2019;36(12):1083-96.	Different phenomenon of interest
81. van Acker, K., et al. (2014). "Burden of diabetic foot disorders, guidelines for management and disparities in implementation in Europe: a systematic literature review." Diabetes Metab Res Rev 30(8): 635-645.	Different study design
82. Villalba C, Jaiprakash A, Donovan J, Roberts J, Crawford R. Unlocking the Value of Literature in Health Co-Design: Transforming Patient Experience Publications into a Creative and Accessible Card Tool. Patient.11(6):637-48.	Different study design
83. von Arx L-B, Kjær T. The Patient Perspective of Diabetes Care: A Systematic Review of Stated Preference Research. The Patient - Patient-Centered Outcomes Research. 2014;7(3):283-300. doi: 10.1007/s40271-014-0057-0.	Different method of evaluation
84. Warshaw H, Hodgson L, Heyman M, Oser TK, Walker HR, Deroze P, et al. The Role and Value of Ongoing and Peer Support in Diabetes Care and Education. Diabetes Educator. 2019;45(6):569-79.	Different phenomenon of interest

85. Welbourn, R., et al. (2016). "A Comparison of Health Professionals' and Patients' Views of the Importance of Outcomes of Bariatric Surgery." PLoS Med 26(11): 2738-2746.	Different study design
86. White, P., et al. (2005). "The role of the family in adult chronic illness: A review of the literature on type 2 diabetes." The Irish Journal of Psychology 26(1-2): 9-15.	Different study design
87. Winkley K, Upsher R, Polonsky WH, Holmes-Truscott E. Psychosocial aspects and contributions of behavioural science to medication-taking for adults with type 2 diabetes. Diabetic Medicine. 2020;37(3):427-35.	Different method of evaluation
88. Wit M, Trief PM, Huber JW, Willaing I. State of the art: understanding and integration of the social context in diabetes care. Diabetic Medicine. 2020;37(3):473-82.	Different study design
89. Xie LF, Itzkovitz A, Roy-Fleming A, Da Costa D, Brazeau AS. Understanding Self-Guided Web-Based Educational Interventions for Patients With Chronic Health Conditions: Systematic Review of Intervention Features and Adherence. J Med Internet Res.22(8):e18355.	Different method of evaluation
90. Yang J, Yang H, Wang Z, Wang X, Wang Y, Yu X, et al. Self-management among type 2 diabetes patients via the WeChat application: A systematic review and meta-analysis. J Clin Pharm Ther.	Different method of evaluation
91. Zhong, Y., et al. (2015). "Cost-utility analyses in diabetes: A systematic review and implications from real-world evidence." Value in Health 18(2): 308-314.	Different phenomenon of interest
92. Zhou T, Guan H, Yao J, Xiong X, Ma A. The quality of life in Chinese population with chronic non-communicable diseases according to EQ-5D-3L: a systematic review. Quality of Life Research. 2018;27(11):2799-814. doi: 10.1007/s11136-018-1928-y.	Different method of evaluation
93. Zhu Ling Koh J-L. Literature review: Factors affecting adherence rate to diabetic treatment options in older adults in the world. Singapore Nursing Journal. 2018;45(1):13-9.	Different study design
