# Supplementary material for: Patients’ and informal caregivers’ perspectives on self-management interventions for type 2 diabetes mellitus outcomes: a mixed-methods overview of 14 years of reviews
Source: Arch Public Health. 2023 Aug 4;81:140. doi: 10.1186/s13690-023-01153-9 (PMC10401891; doi:10.1186/s13690-023-01153-9)
Supplement: Supplementary file 4 — Additional file 4: General characteristics of included SRs [file 13690_2023_1153_MOESM4_ESM.pdf]

Additional file 4

Patients' and informal caregivers’ perspectives on Self-Management Interventions for Type 2 Diabetes Mellitus outcomes: a mixed-methods overview of 14 years of reviews

General characteristics of included SRs

Author (year) Country	Search data frame	Country of included studies	Design and synthesis methods	Phenomena of interest	N studies	N Participants	Main population characteristics	Quality appraisal methods and results
**Albanese 2019(1), USA**	Inception to 2016 (October)	Most from USA	Mixed methods review, Narrative synthesis	The role of spousal support for dietary adherence in T2DM	28	46396	Diverse origin, including patients and patients' caregivers	Not assessed
**Almutairi 2015 (2), Saudi Arabia**	Not specified	Saudi Arabia	Systematic review, Narrative synthesis	Quality of diabetes management in Saudi Arabia	11	Not reported	Comorbidites acute Coronary Syndrome (1), older patients (1), explicit T2DM (3), only women (1)	Russell and Gregory criteria. All studies with 4-5/5 scores
**Bennich 2017 (3), Denmark**	2000-2016	USA (4), and Greece (1)	Mixed methods review, content analysis	Supportive and non-supportive interactions in families	5	614	Patients with T2DM and their partners	CASP were non-comparable
**Biernatzki 2018 (4), Germany**	up to June 2015	Not reported.	Mixed methods review, content analysis	Information needs	25	4641	T1DM and T2DM patients	NICE checklist, Mixed Methods Appraisal Tool three of the 25 studies met all or most of the NICE checklist criteria. In qualitative

								studies, most criteria were fulfilled
**Chen 2015 (5), Taiwan**	2004-2013	UK, Netherlands, Canada, Malaysia	Qualitative evidence synthesis, content analysis	Experiences in self-monitoring of blood glucose	7	190	Adult patients with Type 1 or T2DM	JBI-QARI results not reported
**Coffey 2019 (6), Ireland**	Inception to 2016	Most from UK	Qualitative evidence synthesis, Meta-ethnography	Experiences in foot care and foot ulceration	42	648	T1DM or T2DM participants with previous or current diabetes-related foot complications	CASP, quality was good overall but varied considerably across papers, with scores ranging from 10 to 21 of a possible 24 (M = 16.43, SD = 2.81, median = 17).
**Davies 2013 (7), UK**	up to 2011	Most (13) were conducted exclusively in the USA	Systematic review, Narrative synthesis	Factors affecting adherence to insulin therapy	17	919941	Adults with T1DM or T2DM, most in insulin therapy	A shortened version of (EPHPP) Quality Assessment Tool. Overall, the studies were judged as being of poor quality,
**Ellis 2018 (8), UK**	up to 2015	Asia, Australia, Europe, New Zealand and North America, South America, South Africa	Mixed methods review, Thematic synthesis	Perceptions of insulin use in primary care	34 (23 patients, six with HCPs, and five	13649	Adults with T2DM on the use of insulin	CASP or Barley's checklist, qualitative studies were generally good or

					HCPs and patients)			of moderate strength
**Fleming 2009 (9), UK**	Inception until "present."	Not specified, but ten studies included South Asian migrants living in the UK, and 1 included Kerala people living in India	Qualitative evidence synthesis, Meta-synthesis (theory explication)	Culture, Diabetes, and Nursing in the South Asian Community	11	408	Patients with T1DM or T2DM, first or second-generation South Asians, mainly from Pakistan, India and Bangladesh. Primary studies conducted primarily in the UK	Not assessed
**Foss 2016 (10) , Norway**	2004-2014	UK (10), Sweden (4), Norway (1), Switzerland (1), Netherlands (1), Denmark (1), Germany (1), Belgium (1)	Qualitative evidence synthesis, Meta-ethnography	Connectivity, contest and the ties of SM support	29	540	Patients with T2DM, most from the UK	Campbell et al., (criteria for inclusion)
**Franklin 2018 (11), Australia**	2004-2015	USA (4), Germany (2), Sweden (2), Australia (1), Oman (1), Norway (1), Finland (1), UK (1), Netherlands (1)	Qualitative evidence synthesis, Thematic synthesis	Perceptions of SM support interactions	14	394	Adult patients with T2DM, most from Europe, especially from the UK	CASP, criteria for inclusion
**Frost 2014 (12), UK**	2000-2013	UK (22)	Qualitative evidence synthesis, Meta-ethnography	Diabetes SM strategies for long term medical outcomes and quality of life in the UK	40	184	Adults with T2DM, studies conducted in the UK	Wallace et al. quality tool
**Gorst 2019 (13), UK**	Inception to 2017	Mostly from USA and UK	Qualitative evidence synthesis, content analysis	Patients' perspectives on the initial stages of COS development	26	976	people with T2DM	CASP, the majority were good or fair

**Graham-Rowe 2018 (14), UK**	1990-2017	43% USA; 13% UK; and others	Mixed methods review, Framework synthesis (Theoretical Domains Framework)	Barriers to and enablers of diabetic retinopathy screening attendance	69	2532466	people with T1DM or T2DM	CASP and the Mixed Methods Appraisal Tool majority were at low (46.7%), or unclear (45%) risk of bias
**Ho 2010 (15), Sweden**	Since 2000	UK	Qualitative evidence synthesis, Meta-ethnography	Diabetes empowerment related to Pender's Health Promotion Model	9	197	Patients with T1DM or T2 DM, some from Pakistan or South Asians in UK.	Not assessed
**Horigan 2017 (16), UK**	2005-2015	Europe, USA, Pakistan, Canada and India	Mixed methods review, Thematic synthesis	Reasons why patients referred to diabetes education programmes choose not to attend	12	2260	Patients with T2DM not-attending education programmes	Not assessed
**Inga-Britt 2018 (17), Sweden**	2007-2011	Mainly USA and less frequently in Europe and Australia	Qualitative evidence synthesis, Meta-synthesis (interpretive constructivist approach)	Challenges faced in daily life by persons with T2DM	37	884	persons with T2DM	Not assessed
**Jain 2020 (18), Singapore**	Inception to August 2019	United States (5), United Kingdom (5), Canada (3), Iran (1), Belgium (1)	Qualitative evidence synthesis, Thematic synthesis	The features, use, and implementation of technology-assisted Diabetes Self Management Education	15	242	Patients with T2DM and HCP ranged from 18 to 81 years. SMIs: Web-based, mobile phone application, digital versatile disc, virtual reality, telehealth.	CASP: no or very minor concerns=8/15; minor concerns=3/15; moderate concerns=4/15

**Jalil 2015 (19), Australia**	Not specified	USA, UK, South Korea, Australia	Systematic review, Meta-synthesis	Behavioural Outcomes from Telemedicine Clinical Trials	19	2979	people with T2DM	Not assessed
**Jones 2017 (20), New Zealand**	1946-2016	USA (21), UK (3), Australia (1), Sweden (1), Belgium (1)	Qualitative evidence synthesis, Thematic synthesis	Ethnic minorities' experiences of management of T2DM	27	946	Patients with T2DM from ethnic minorities (African American, Hispanic, Latino, Chinese and Korean)	CAS, GRADE-CERQual for certainty (2 moderate and three high confidence)
**Joo 2021 (21) , Korea**	2009-2019	USA	Qualitative evidence synthesis, Thematic synthesis	Experiences and perceptions that ethnic minorities in the United States with T2DM had about culturally tailored interventions	7	201	Ethnic minorities with T2DM living in USA, including immigrants and born in the USA	CASP: all studies considered to be of high quality
**Kashim 2018 (22), UK**	2013-2017	15 from UK and one each from Ireland, Iceland, The Netherlands, Saudi Arabia and USA	Mixed methods review, Vote counting/Thematic synthesis	Patients' Non-Attendance to diabetic retinopathy screening	16	126888	T1DM and T2DM	McMaster critical appraisal tool **(criteria for inclusion)**
**Kumar 2016 (23), UK**	1980-2015	UK	Qualitative evidence synthesis, Thematic analysis and grounded theory	Adherence-related beliefs about medicine amongst patients of South Asian origin with diabetes and cardiovascular disease	21	416	UK South Asian patients were taking diabetic medicines for T2DM or cardiovascular diseases.	Not assessed
**Li 2014 (24), Australia**	1990-2010	USA, Australia, and others not reported.	Qualitative evidence synthesis, Meta-aggregation	The experience of older women living and coping with T2DM	9	Not reported	women > 18 years with a diagnosis T2DM from USA, Australia	JBI QARI, Four/41 findings were excluded

**Li-Geng 2020 (25) , USA**	1995-2018	USA	Mixed methods review, Thematic synthesis	EEAs perspectives and experiences with dietary SM of diabetes	16	261	Participants from China and Korea spoke their native language and English. The average age ranged from 55 to 68, and the average time living in the USA ranged from 14 to 25 years. The majority were male (57%)	Pluye's mixed methods appraisal tool. RCT 2 , both 75% (due to no blinding); Controlled cohort study 1, 100%, single cohort 3, 100%(2), and one 75%
**Madsen 2019 (26), Denmark**	Inception to 2018	Not reported	Systematic review, Narrative synthesis	Associations between time preferences, diabetes SM behaviours and outcomes.	13	41265	Half of the studies included only patients with T2DM (6), four did not report the type of DM, 3 included T1DM	The general assessment tool, quality varied,
**Maine 2018 (27), UK**	Not specified	New Zeland, Australia, UK	Qualitative evidence synthesis, Meta-aggregation	Diabetes SM in adults with intellectual disabilities and their caregivers	8	177	Adults with T2DM and intellectual disabilities and their caregivers	The appraisal was reported of rigour
**Majeed-Ariss 2015 (28), UK**	1986-2008	USA (38), UK (11), Canada (3), Scandinavia (3), Australia (2)	Mixed methods review, Thematic synthesis	Black and ethnic minority patients' views on SM of T2DM	57	1723	Patients with T2DM black and ethnic minorities living in the USA, UK, and Australia. Africans and Latinos were the largest	Adaptation of Long and Godfrey and the EPPI Centre's review guidelines. Seven scored poorly.
**Matricciani 2015 (29), Australia**	Not reported. Apparently , up to 2013	US (5), UK (2), Australia (1), Germany (1)	Mixed methods review, Thematic synthesis	Foot self-care practices among older adults diagnosed with diabetes	9	4841	Non-institutionalised older adults diagnosed with T1DM or T2DM	STROBE statement, CASP, eight quantitative studies varied

								between 15 and 20 (out of 22)
**Miller 2018 (30), USA**	up to 2017	Europe, North America, Australia, and Asia	Qualitative evidence synthesis, Meta-synthesis (critical realistic approach)	Factors influencing participation in physical activity after dysvascular amputation	14	353	Patients with T2DM who experienced a dysvascular lower limb amputation from urban and suburban environments	McMaster University's Guidelines for Qualitative Review, variable methodological quality
**Nam 2011 (31) USA**	1990-2009	Most from USA	Mixed methods review, Narrative synthesis	Barriers to diabetes management from the perspectives of both patients and clinicians	80	58145	Patients with T2DM and their providers (only patients' findings extracted).	Not assessed
**Ng 2015 (32) Malaysia**	up to 2014	UK, USA, Malaysia, Singapore, South Africa, Australia, Belgium, Canada, Israel, Japan, Romania USA and Canada Arabic countries	Mixed methods review, Narrative synthesis	Barriers and facilitators to insulin initiation	25	21881	Patients with T2DM who are insulin naıve	QualSyst, most were of good quality.
**Peimani 2018 (33) Iran**	2000-2017	Iran	Mixed methods review, Narrative synthesis	Barriers and facilitators to effective communication from the patient's perspective	22	26720	Patients with T2DM	JBI Critical Appraisal Checklists, the quality of most studies was moderate to high based

**Pennbrant 2019 (34) Sweden**	2007–2018	USA (5), Australia (1), China (1), Korea (1), UK (1)	Qualitative evidence synthesis, Evans's analysis model	The experience of older patients with diabetes self-care	9	256	Older people with diabetes (65 years or older)	Critical Appraisal Skills Programme, 2/9 were considered as medium quality. The remaining were considered as high
**Polinski 2013 (35) USA**	Inception to 2011	Canada (1), USA (2), from different countries (1), Non reported (2)	Systematic review, Narrative synthesis	barriers to insulin progression	6	16199	T2DM (5), T1DM or T2DM (2), 100% insulin (2); 44%-49% insulin users(3), 100% inadequately controlled T2DM (1)	9-point, modified assessment checklist, ≥5 (3), 1-4 (3)
**Psarou 2018 (36) UK**	Inception to 2016	UK region	Qualitative evidence synthesis, Meta-ethnography	Patients' journeys in medication management, the perspective of oral and injectable medicines, body weight and medicine-taking behaviour	16	360	Patients with T2DM are mostly of Caucasian and South Asian origin, all of whom were recruited from different UK region	CASP articles were only included if they scored ≥ 7
**Pun 2009 (37) China**	1986-2007	USA, UK, multiple countries	Mixed methods review, Framework synthesis (Health Belief Model)	Barriers to diabetes self-care	16	8959	Patients with T1DM or T2DM (mostly T2DM) from worldwide, mainly from the USA	Not assessed
**Rai 2020 (38)**<br><br>**The Netherlands**	Not specified	Most were high-income countries USA, Australia, Japan, Korea, UK, Norway, Switzerland, and Singapore. India,	Mixed methods review, Thematic synthesis	The stigma associated with the top four non-communicable diseases	26 (7 Diabetes, 2 T1DM, 5 T2DM)	3733 (Type 2DM)	T2DM % male in a range from 54.8% and 80.4%	14-item criteria for quantitative and 10-item criteria for qualitative studies. Only one

		Ghana, Uganda, and Thailand.						scored 0.8; others 0,60 to 0,67
**Rouyard 2017 (39) UK**	2002-2014	USA, UK, The Netherlands, Australia, Pakistan, Spain	Mixed methods review, Narrative synthesis	Lay risk perceptions of complications of patients with T2DM	18	4696	Patients with T2DM, most without cardiovascular disease, some were recently diagnosed, mainly from the USA, and the UK	CASP and Quality Assessment Tool (QAT). Two/4 were good. Results were mixed for quantitative studies.
**Sarayani 2013 (40) Iran**	up to July 2012	Iran	Mixed methods review, Narrative synthesis	Adherence to diabetes medications in Iranian population with T2DM	14	2658	Patients with T2DM medications from Iran	Not explicit, Methodological caveats in some studies.
**Scarton 2014 (41) USA**	1990-2013	Europe, USA and South Africa	Mixed methods review, Framework synthesis (Cross-cultural Literature)	Family caregivers needs	69	361	Family caregivers of persons with T2DM	Not assessed
**Schmidt-Busby 2018 (42)**<br>**New Zealand**	2000-2016	Australia, Canada, New Zealand, the UK, the USA, and Western Europe. Most studies were conducted in the USA.	Mixed methods review, Framework synthesis (Realist approach)	Contextual factors affecting SM interventions for T2DM with co-morbidities	30	1938	Patients with T2DM with co-morbidities	CASP, NICE checklist, results not reported (inclusion criteria)
**Sibounheuang 2020 (43) Thailand**	2001-2017	USA, Canada, Netherland Oman(and Taiwan, Japan, Malaysia, Iran, Australia, Germany, Finland,	Qualitative evidence synthesis, Thematic synthesis	Patients and health care providers' contexts of diabetes management	23 (18 patients with T2DM)	500	T2DM patients 2/18 included patients older than 60 y	CASP checklist 11/18 =8 to 10/10points

		UK, America Samoa, and Bangladesh						
**Singh 2016 (44) Australia**	1980-2015	Australia, USA, the Netherlands, Denmark, Poland and UK.	Systematic review, Narrative synthesis	Perceptions of telehealth for treatment of foot ulcer	11	380	Patients with diabetic foot ulcers had to have telehealth-guided management	Not assessed
**Sohal 2015 (45) Canada**	1990-2014	India, the UK, Norway and the USA	Qualitative evidence synthesis, Meta-ethnography	Barriers and facilitators to diabetes management South Asian patients	20	1980	South Asian T2DM, The majority were women aged between 20–80 years.	CASP, NIH quality assessment tool; Good and fair studies,
**Spenceley 2006 (46) Canada**	1993-2003	USA (10), Canada (3), UK (2), New Zealand (1)	Mixed methods review (critical integrative review), Narrative synthesis	Self-care from the perspective of patients	16	Not reported	Patients with either T1DM or T2DM mostly	Specific tools adapted from several authors<br><br>(Criteria for inclusion)
**Stiffler 2014 (47)USA**	2005-2012	Ireland, USA, Australia, New Zeland, UK, Netherlands, Finland, Denmark, Norway, Scotland, Pakistan, Iran, Belgium	Qualitative evidence synthesis, Meta-aggregation	Patients' experiences within the context of diabetes self-care while facing daily barriers	21	730	T1DM or T2DM patients	JBI QARI, criteria for inclusion.
**Suglo 2020 (48) USA**	2000-2019	WHO Africa Region	Qualitative evidence synthesis, Thematic synthesis	Diabetes SM	16	423	T2DM patients, adults above 18 years of age, majority of the participants were females (n = 271, 64%)	JBI-QARI, The majority of the included studies were of sound methodological quality.

**Vanstone 2017 (49) Canada**	2002-2015	North America, Europe, Australia, or New Zealand	Qualitative evidence synthesis, Meta-synthesis	Barriers to diet modification	120	3721	Marginalised and non-marginalised adults with T2DM	Not assessed
**Verhoeven 2007 (50) The Netherlands**	1994-2006	developed countries, mainly from Europe.	Systematic review, Narrative synthesis	Benefits and deficiencies of teleconsultation and videoconferencing regarding clinical, behavioural, and care coordination outcomes of diabetes care	39	4809	Patients with T1, T2DM or gestational diabetes using teleconsultation and videoconferencing	Jadad scale (for 8 RCTs), The Jadad quality score of the trials was either 2 or 3
**Vongmany 2018 (51)**<br><br>**Australia**	2000-2016	More than half were conducted in high-income countries	Qualitative evidence synthesis, Thematic synthesis	Family behaviours that have an impact on adults living with T2DM SM practices	40	928	Patients with T2DM. Mostly women. Half focused on culturally and linguistically diverse or disadvantaged populations.	CASP, the quality of studies was variable.
**Walker 2019 (52) New Zealand**	Inception to 2017	Scotland/England, Taiwan	Qualitative evidence synthesis, Thematic synthesis	Patients' beliefs, attitudes, expectations, and experiences of remote monitoring.	16 (4 diabetes)	83 (Diabetes)	3/4 were in patients who have experienced telemonitoring, and two were explicitly for T2DM, 1 in patients older than 65y, one in patients aged 23 to 92y, and 2 non-reported.	COREQ framework, 2 studies had 20/25(80%= items reported, and 2, 17/25 (68%)
**Wang 2012 (53)Taiwan**	1999-2009	Most developed countries	Mixed methods review, Thematic synthesis	Psychological resistance to insulin therapy from the perspective of adults with T2DM	16	7961	Adults with T2DM	CASP, a tool adapted from Lethaby et al .6/ 16 were considered as poor quality (+)

**Zeh 2014 (54) UK**	Inception to 2011	Most from UK	Mixed methods review, Thematic synthesis	Cultural barriers to accessing and using diabetes services	22	1827	T1DM or T2DM patients from ethnic minorities living in Western countries (most from the UK)	Popay et al Jadad et al and Creswell and Plano Clark. Fourteen studies were of good quality, and eight were of moderate quality.

1. Albanese AM, Huffman JC, Celano CM, Malloy LM, Wexler DJ, Freedman ME, et al. The role of spousal support for dietary adherence among type 2 diabetes patients: a narrative review. Soc Work Health Care. 2019;58(3):304-23.
2. Almutairi KM. Quality of Diabetes Management in Saudi Arabia: A Review of Existing Barriers. Arch Iran Med. 2015;18(12):816-21.
3. Bennich BB, Roder ME, Overgaard D, Egerod I, Munch L, Knop FK, et al. Supportive and non-supportive interactions in families with a type 2 diabetes patient: an integrative review. Diabetol Metab Syndr. 2017;9:57.
4. Biernatzki L, Kuske S, Genz J, Ritschel M, Stephan A, Bachle C, et al. Information needs in people with diabetes mellitus: a systematic review. Syst Rev. 2018;7(1):27.
5. Chen CM, Chang Yeh M. The experiences of diabetics on self-monitoring of blood glucose: a qualitative metasynthesis. J Clin Nurs. 2015;24(5-6):614-26.
6. Coffey L, Mahon C, Gallagher P. Perceptions and experiences of diabetic foot ulceration and foot care in people with diabetes: A qualitative meta-synthesis. Int Wound J. 2019;16(1):183-210.
7. Davies MJ, Gagliardino JJ, Gray LJ, Khunti K, Mohan V, Hughes R. Real-world factors affecting adherence to insulin therapy in patients with Type 1 or Type 2 diabetes mellitus: a systematic review. Diabet Med. 2013;30(5):512-24.
8. Ellis K, Mulnier H, Forbes A. Perceptions of insulin use in type 2 diabetes in primary care: a thematic synthesis. BMC Fam Pract. 2018;19(1):70.
9. Fleming E, Gillibrand W. An exploration of culture, diabetes, and nursing in the South Asian community: a metasynthesis of qualitative studies. J Transcult Nurs. 2009;20(2):146-55.
10. Foss C, Knutsen I, Kennedy A, Todorova E, Wensing M, Lionis C, et al. Connectivity, contest and the ties of self-management support for type 2 diabetes: a meta-synthesis of qualitative literature. Health Soc Care Community. 2016;24(6):672-86.
11. Franklin M, Lewis S, Willis K, Bourke-Taylor H, Smith L. Patients' and healthcare professionals' perceptions of self-management support interactions: Systematic review and qualitative synthesis. Chronic Illn. 2018;14(2):79-103.
12. Frost J, Garside R, Cooper C, Britten N. A qualitative synthesis of diabetes self-management strategies for long term medical outcomes and quality of life in the UK. BMC Health Serv Res. 2014;14:348.
13. Gorst SL, Young B, Williamson PR, Wilding JPH, Harman NL. Incorporating patients' perspectives into the initial stages of core outcome set development: a rapid review of qualitative studies of type 2 diabetes. BMJ Open Diabetes Res Care. 2019;7(1):e000615.
14. Graham-Rowe E, Lorencatto F, Lawrenson JG, Burr JM, Grimshaw JM, Ivers NM, et al. Barriers to and enablers of diabetic retinopathy screening attendance: a systematic review of published and grey literature. Diabet Med. 2018;35(10):1308-19.
15. Ho AY, Berggren I, Dahlborg-Lyckhage E. Diabetes empowerment related to Pender's Health Promotion Model: a meta-synthesis. Nurs Health Sci. 2010;12(2):259-67.
16. Horigan G, Davies M, Findlay-White F, Chaney D, Coates V. Reasons why patients referred to diabetes education programmes choose not to attend: a systematic review. Diabet Med. 2017;34(1):14-26.
17. Inga-Britt L, Kerstin B. Challenges faced in daily life by persons with type 2 diabetes: A meta-synthesis. Chronic Illn. 2018:1742395318757852.
18. Jain SR, Sui Y, Ng CH, Chen ZX, Goh LH, Shorey S. Patients' and healthcare professionals' perspectives towards technology-assisted diabetes self-management education. A qualitative systematic review. PLoS One. 2020;15(8):e0237647.
19. Jalil S, Myers T, Atkinson I. A meta-synthesis of behavioral outcomes from telemedicine clinical trials for type 2 diabetes and the Clinical User-Experience Evaluation (CUE). J Med Syst. 2015;39(3):28.
20. Jones V, Crowe M. How people from ethnic minorities describe their experiences of managing type-2 diabetes mellitus: A qualitative meta-synthesis. Int J Nurs Stud. 2017;76:78-91.

21. Joo JY, Liu MF. Experience of Culturally-Tailored Diabetes Interventions for Ethnic Minorities: A Qualitative Systematic Review. Clin Nurs Res. 2021;30(3):253-62.
22. Kashim RM, Newton P, Ojo O. Diabetic Retinopathy Screening: A Systematic Review on Patients' Non-Attendance. Int J Environ Res Public Health. 2018;15(1).
23. Kumar K, Greenfield S, Raza K, Gill P, Stack R. Understanding adherence-related beliefs about medicine amongst patients of South Asian origin with diabetes and cardiovascular disease patients: a qualitative synthesis. BMC Endocr Disord. 2016;16(1):24.
24. Li J, Drury V, Taylor B. A systematic review of the experience of older women living and coping with type 2 diabetes. Int J Nurs Pract. 2014;20(2):126-34.
25. Li-Geng T, Kilham J, McLeod KM. Cultural Influences on Dietary Self-Management of Type 2 Diabetes in East Asian Americans: A Mixed-Methods Systematic Review. Health Equity. 2020;4(1):31-42.
26. Madsen KP, Kjaer T, Skinner T, Willaing I. Time preferences, diabetes self-management behaviours and outcomes: a systematic review. Diabet Med. 2019;36(11):1336-48.
27. Maine A, Brown M, Dickson A, Truesdale M. The experience of type 2 diabetes self-management in adults with intellectual disabilities and their caregivers: A review of the literature using meta-aggregative synthesis and an appraisal of rigor. J Intellect Disabil. 2018:1744629518774172.
28. Majeed-Ariss R, Jackson C, Knapp P, Cheater FM. A systematic review of research into black and ethnic minority patients' views on self-management of type 2 diabetes. Health Expect. 2015;18(5):625-42.
29. Matricciani L, Jones S. Who cares about foot care? Barriers and enablers of foot self-care practices among non-institutionalized older adults diagnosed with diabetes: an integrative review. Diabetes Educ. 2015;41(1):106-17.
30. Miller MJ, Jones J, Anderson CB, Christiansen CL. Factors influencing participation in physical activity after dysvascular amputation: a qualitative meta-synthesis. Disabil Rehabil. 2018:1-10.
31. Nam S, Chesla C, Stotts NA, Kroon L, Janson SL. Barriers to diabetes management: patient and provider factors. Diabetes Res Clin Pract. 2011;93(1):1-9.
32. Ng CJ, Lai PS, Lee YK, Azmi SA, Teo CH. Barriers and facilitators to starting insulin in patients with type 2 diabetes: a systematic review. Int J Clin Pract. 2015;69(10):1050-70.
33. Peimani M, Nasli-Esfahani E, Sadeghi R. Patients' perceptions of patient-provider communication and diabetes care: A systematic review of quantitative and qualitative studies. Chronic Illn. 2018:1742395318782378.
34. Pennbrant S, Berg A, Fohlin Johansson L. Self-care experiences of older patients with diabetes mellitus: A qualitative systematic literature review. Nordic Journal of Nursing Research. 2020;40(2):64-72.
35. Polinski JM, Smith BF, Curtis BH, Seeger JD, Choudhry NK, Connolly JG, et al. Barriers to insulin progression among patients with type 2 diabetes: a systematic review. Diabetes Educ. 2013;39(1):53-65.
36. Psarou A, Cooper H, Wilding JPH. Patients' Perspectives of Oral and Injectable Type 2 Diabetes Medicines, Their Body Weight and Medicine-Taking Behavior in the UK: A Systematic Review and Meta-Ethnography. Diabetes Ther. 2018;9(5):1791-810.
37. Pun S, Coates V, Benzie I. Barriers to the self-care of type 2 diabetes from both patients' and providers' perspectives: literature review. J Nurs Healthc Chronic Illn. 2009;1:4e19.
38. Rai SS, Syurina EV, Peters RMH, Putri AI, Zweekhorst MBM. Non-Communicable Diseases-Related Stigma: A Mixed-Methods Systematic Review. Int J Environ Res Public Health. 2020;17(18).
39. Rouyard T, Kent S, Baskerville R, Leal J, Gray A. Perceptions of risks for diabetes-related complications in Type 2 diabetes populations: a systematic review. Diabet Med. 2017;34(4):467-77.
40. Sarayani A, Jahangard-Rafsanjani Z, Hadjibabaie M, Ahmadvand A, Javadi M, Gholami K. A comprehensive review of adherence to diabetes and cardiovascular medications in Iran; implications for practice and research. J Diabetes Metab Disord. 2013;12(1):57.

41. Scarton LJ, Bakas T, Miller WR, Poe GD, Huber LL. Needs and Concerns of Family Caregivers of Persons With Type 2 Diabetes: An Integrated Review of Cross-cultural Literature With Implications for the American Indian Population. Diabetes Educ. 2014;40(4):444-52.
42. Schmidt-Busby J, Wiles J, Exeter D, Kenealy T. Understanding 'context' in the self-management of type 2 diabetes with comorbidities: A systematic review and realist evaluation. Diabetes Res Clin Pract. 2018;142:321-34.
43. Sibounheuang P, Olson PS, Kittiboonyakun P. Patients' and healthcare providers' perspectives on diabetes management: A systematic review of qualitative studies. Res Social Adm Pharm. 2020;16(7):854-74.
44. Singh TP, Vangaveti VN, Kennedy RL, Malabu UH. Role of telehealth in diabetic foot ulcer management - A systematic review. Aust J Rural Health. 2016;24(4):224-9.
45. Sohal T, Sohal P, King-Shier KM, Khan NA. Barriers and Facilitators for Type-2 Diabetes Management in South Asians: A Systematic Review. PLoS One. 2015;10(9):e0136202.
46. Spenceley SM, Williams BA. Self-care from the perspective of people living with diabetes. Can J Nurs Res. 2006;38(3):124-45.
47. Stiffler D, Cullen D, Luna G. Diabetes barriers and self-care management: the patient perspective. Clin Nurs Res. 2014;23(6):601-26.
48. Suglo JN, Evans C. Factors influencing self-management in relation to type 2 diabetes in Africa: A qualitative systematic review. PLoS One. 2020;15(10):e0240938.
49. Vanstone M, Rewegan A, Brundisini F, Giacomini M, Kandasamy S, DeJean D. Diet modification challenges faced by marginalized and nonmarginalized adults with type 2 diabetes: A systematic review and qualitative meta-synthesis. Chronic Illn. 2017;13(3):217-35.
50. Verhoeven F, van Gemert-Pijnen L, Dijkstra K, Nijland N, Seydel E, Steehouder M. The contribution of teleconsultation and videoconferencing to diabetes care: a systematic literature review. J Med Internet Res. 2007;9(5):e37.
51. Vongmany J, Luckett T, Lam L, Phillips JL. Family behaviours that have an impact on the self-management activities of adults living with Type 2 diabetes: a systematic review and meta-synthesis. Diabet Med. 2018;35(2):184-94.
52. Walker RC, Tong A, Howard K, Palmer SC. Patient expectations and experiences of remote monitoring for chronic diseases: Systematic review and thematic synthesis of qualitative studies. Int J Med Inform. 2019;124:78-85.
53. Wang HF, Yeh MC. Psychological resistance to insulin therapy in adults with type 2 diabetes: mixed-method systematic review. J Adv Nurs. 2012;68(4):743-57.
54. Zeh P. Cultural barriers impeding ethnic minority groups from accessing effective diabetes care services: a systematic review of observational studies. Diversity & Equality in Health & Care. 2014;11(1):9-33.
