# Supplementary material for: Patients’ and informal caregivers’ perspectives on self-management interventions for type 2 diabetes mellitus outcomes: a mixed-methods overview of 14 years of reviews
Source: Arch Public Health. 2023 Aug 4;81:140. doi: 10.1186/s13690-023-01153-9 (PMC10401891; doi:10.1186/s13690-023-01153-9)
Supplement: Supplementary file 5 — Additional file 5: JBI Critical Appraisal Checklist assessment [file 13690_2023_1153_MOESM5_ESM.pdf]

*Additional file 5*

# Patients' and informal caregivers' perspectives on Self-Management Interventions for Type 2 Diabetes Mellitus outcomes: a mixed-methods overview of 14 years of reviews

***JBI Critical Appraisal Checklist assessment***

| *Study ID* | Q1 | Q2 | Q3 | Q4 | Q5 | Q6 | Q7 | Q8 | Q10 | Q11 | SCORE |
|---|---|---|---|---|---|---|---|---|---|---|---|
| *Albanese 2019* | Yes | Yes | Unclear | Unclear | No | No | No | Yes | Yes | Yes | **50%** |
| *Almutairi 2015* | Yes | Yes | Yes | Yes | Yes | Unclear | Yes | Unclear | Yes | Yes | **80%** |
| *Bennich 2017* | Yes | Yes | Yes | Yes | Yes | Unclear | Unclear | Yes | Yes | Yes | **80%** |
| *Biernatzki 2018* | Yes | Yes | Yes | Yes | Yes | Yes | Yes | Yes | Unclear | Yes | **90%** |
| *Chen 2015* | Yes | Yes | Yes | Yes | Yes | Yes | Yes | Yes | Yes | Yes | **100%** |
| *Coffey 2018* | Yes | Yes | Yes | Yes | Yes | Yes | Yes | Yes | Yes | Yes | **100%** |
| *Davies 2013* | Yes | Yes | Yes | Yes | Unclear | Unclear | Yes | Yes | Yes | Yes | **80%** |
| *Ellis 2018* | Yes | Yes | Yes | Yes | Yes | Unclear | Yes | Yes | Yes | Yes | **90%** |
| *Fleming 2009* | Yes | Yes | Unclear | Yes | No | No | Yes | Yes | Yes | Yes | **70%** |
| *Foss 2016* | Yes | Yes | Unclear | Yes | Unclear | No | Yes | Yes | Yes | Yes | **70%** |
| *Franklin 2018* | Yes | Yes | Yes | Yes | Yes | Unclear | Yes | Yes | Yes | Yes | **90%** |
| *Frost 2014* | Yes | Yes | Yes | Yes | Yes | Unclear | Yes | Yes | Yes | Yes | **90%** |
| *Gorst 2019* | Yes | Yes | Yes | Yes | Yes | No | Yes | Unclear | Yes | Yes | **80%** |
| *Graham-Rowe 2018* | Yes | No | Yes | Yes | Yes | Yes | Yes | Yes | Yes | Yes | **90%** |
| *Ho 2010* | Yes | Unclear | No | Yes | No | No | Unclear | Yes | Yes | Yes | **50%** |
| *Horigan 2017* | Yes | Yes | Unclear | Yes | No | No | Yes | Yes | Yes | Yes | **70%** |
| *Inga-Britt 2018* | Yes | Yes | Yes | Yes | No | No | Yes | Yes | Yes | Yes | **80%** |
| *Jain 2020* | Yes | Unclear | Yes | Yes | Yes | Yes | Yes | Yes | Yes | Yes | **90%** |
| *Jalil 2015* | Yes | Yes | Unclear | Yes | No | No | Unclear | Yes | Yes | Yes | **60%** |
| *Jones 2017* | Yes | Yes | Yes | Yes | Yes | Yes | Yes | Yes | Yes | Yes | **100%** |
| *Joo 2019* | Yes | Yes | Yes | Yes | Yes | Yes | Yes | Yes | Yes | Yes | **100%** |
| *Kashim 2018* | Yes | Yes | Yes | Unclear | Yes | Unclear | Unclear | Yes | Yes | No | **60%** |
| *Kumar 2016* | Yes | Yes | Unclear | Yes | No | No | Yes | Yes | Yes | Yes | **70%** |

| | | | | | | | | | | | |
|---|---|---|---|---|---|---|---|---|---|---|---|
| *Li 2014* | Yes | Yes | Unclear | Yes | Yes | Yes | Unclear | Yes | Yes | Yes | **80%** |
| *Li-Geng 2020* | Yes | Yes | Yes | Yes | Yes | Unclear | Unclear | Yes | Yes | Yes | **80%** |
| *Madsen 2019* | Yes | Yes | Yes | Yes | Unclear | Unclear | Unclear | Yes | Yes | Yes | **70%** |
| *Maine 2018* | Yes | Yes | Yes | Yes | Unclear | Yes | Unclear | Yes | Yes | Yes | **90%** |
| *Majeed-Ariss 2015* | Yes | Yes | Yes | Yes | Unclear | Yes | Yes | Yes | Yes | Yes | **90%** |
| *Matricciani 2015* | Yes | Yes | Yes | Yes | Yes | Unclear | Unclear | Yes | Yes | Yes | **80%** |
| *Miller 2018* | Yes | Yes | Unclear | Yes | Yes | Yes | Yes | Yes | Yes | Yes | **90%** |
| *Nam 2011* | Yes | Unclear | Yes | Yes | No | No | Unclear | Yes | Yes | Yes | **60%** |
| *Ng 2015* | Yes | Yes | Yes | Yes | Yes | Yes | Yes | Yes | Yes | Yes | **100%** |
| *Peimani 2018* | Yes | Yes | Yes | Yes | Yes | Yes | Unclear | Yes | Yes | Yes | **90%** |
| *Pennbrant 2019* | Yes | Yes | Yes | Yes | Yes | Yes | Yes | Yes | Yes | Yes | **100%** |
| *Polinski 2013* | Yes | Yes | Yes | Yes | Unclear | Yes | Yes | Yes | Yes | Yes | **100%** |
| *Psarou 2018* | Yes | Yes | Yes | Yes | Yes | Yes | Unclear | Yes | Yes | Yes | **90%** |
| *Pun 2009* | Yes | Yes | Yes | Yes | No | No | Unclear | Unclear | Yes | Unclear | **50%** |
| *Rai 2020* | Yes | Yes | Yes | Yes | Unclear | Yes | Yes | Yes | Yes | Yes | **90%** |
| *Rouyard 2017* | Yes | Yes | Yes | Yes | Yes | Yes | Unclear | Yes | Yes | Yes | **90%** |
| *Sarayani 2013* | Yes | Yes | Yes | Yes | Unclear | Yes | Yes | Yes | Yes | Yes | **90%** |
| *Scarton 2014* | Yes | Yes | Yes | Yes | No | No | Unclear | Yes | Yes | Yes | **70%** |
| *Schmidt-Busby 2018* | Yes | Yes | Yes | Yes | Yes | Unclear | Yes | Yes | Yes | Yes | **90%** |
| *Sibounheuang 2020* | Yes | Yes | Yes | Yes | Yes | Yes | Yes | Yes | Yes | Yes | **100%** |
| *Singh 2016* | Yes | Yes | Yes | Yes | No | No | Yes | Yes | Yes | Yes | **80%** |
| *Sohal 2015* | Yes | Yes | Yes | Yes | Yes | Unclear | Yes | Yes | Yes | No | **80%** |
| *Spenceley 2006* | Yes | Yes | No | Yes | Unclear | Yes | Unclear | Unclear | Yes | Yes | **60%** |
| *Stiffler 2014* | Yes | Yes | Unclear | Yes | Yes | Yes | Yes | Yes | Yes | No | **80%** |
| *Suglo 2020* | Yes | Yes | Yes | Yes | Yes | Yes | Yes | Yes | Yes | Yes | **100%** |
| *Vanstone 2017* | Yes | Yes | Yes | Yes | No | No | Yes | Yes | Yes | No | **70%** |
| *Verhoeven 2007* | Yes | Yes | Yes | Yes | Unclear | Unclear | Yes | Yes | Yes | Yes | **80%** |
| *Vongmany 2018* | Yes | Yes | Yes | Yes | Yes | No | Unclear | Yes | Yes | Yes | **80%** |
| *Walker 2019* | Yes | Yes | Yes | Yes | Unclear | Unclear | Yes | Yes | Yes | Yes | **80%** |
| *Wang 2012* | Yes | Yes | Unclear | Yes | Yes | Yes | Yes | Yes | Yes | Yes | **90%** |
| *Zeh 2014* | Yes | Yes | Yes | Yes | Yes | Unclear | Yes | Yes | Yes | Yes | **90%** |

Q1. Is the review question clearly and explicitly stated?
Q2. Were the inclusion criteria appropriate for the review question?
Q3. Was the search strategy appropriate?
Q4. Were the sources and resources used to search for studies adequate?
Q5. Were the criteria for appraising studies appropriate?
Q6. Was critical appraisal conducted by two or more reviewers independently?"
Q7. Were there methods to minimise errors in data extraction?
Q8. Were the methods used to combine studies appropriate?
Q9. Was the likelihood of publication bias assessed? (not applicable in this set of studies)
Q10. Were recommendations for policy and/or practice supported by the reported data?
Q11. Were the specific directives for new research appropriate**?**
