# Supplementary material for: Patients’ and informal caregivers’ perspectives on self-management interventions for type 2 diabetes mellitus outcomes: a mixed-methods overview of 14 years of reviews
Source: Arch Public Health. 2023 Aug 4;81:140. doi: 10.1186/s13690-023-01153-9 (PMC10401891; doi:10.1186/s13690-023-01153-9)
Supplement: Supplementary file 6 — Additional file 6: Overlap analysis [file 13690_2023_1153_MOESM6_ESM.pdf]

Additional file 6

Patients' and informal caregivers' perspectives on Self-Management Interventions for Type 2 Diabetes Mellitus outcomes: a mixed-methods overview of 14 years of reviews

Overlap analysis

Overall results		
Number of columns (number of reviews)	c	54
Number of rows (number of index publications)	r	939
r of included primary studies (including double counting)	N	1314
Covered area	N/(rc)	2.59%
Corrected covered area	(N-r)/(rc-r)	0.75%
Interpretation of overlap	Slight overlap	
Structural Zeros	X	0
Corrected covered area (adjusting by structural zeros)	(N-r)/(rc-r-X)	0.75%
N° of non-overlapped primary studies	In 1 SR	748
Number of overlapped primary studies	In 2 SRs	126
	In 3 SRs	40
	In 4 SRs	20
	In 5 SRs	2
	In 6 SRs	0
	In 7 SRs	2
	In 8 SRs	0
	In 9 SRs	0
	In 10 SRs	0
	In 11 SRs	0
	In 12 SRs	0
	In 13 SRs	0
	In 14 SRs	0
	In 15 or more SRs	1

1431	=	Total nodes (pairs of reviews)
1383	=	Slight overlap (<5%)
39	=	Moderate overlap (5% to <10%)
3	=	High overlap (10% to <15%)
6	=	Very High overlap (≥15%)

	Almutairi 2015	Bennich 2017	Biernatzki 2018	Chen 2015	Coffey 2018	Davies 2013	Ellis 2018	Fleming 2009	Foss 2016	Franklin 2018	Frost 2014	Gorst 2019	Graham-Rowe 2018	Ho 2010	Horigan 2017	Inga-Britt 2018	Jalil 2015	Jones 2017	Kashim 2018	Kumar 2016	Li 2014	Maine 2018	Majeed-Ariss 2015	Matricciani 2015	Miller 2018	Ng 2015	Peimani 2018	Polinski 2013	Psarou 2018	Pun 2009	Rouyard 2017	Sarayani 2013	Scarton 2014	Schmidt-Busby, 2018	Singh 2016	Sohal 2015	Spenceley 2006	Stiffler 2014	Vanstone 2017	Verhoeven 2007	Vongmany 2018	Zeh 2014	Nam 2011	Wang 2012	Albanese 2019	Jain 2020	Joo 2021	Li-Geng2020	Madsen 2019	Pennbrant 2020	Rai 2020	Sibounheuang 2020	Suglo 2020
Bennich 2017	0.0%
Biernatzki 2018	0.0%	0.0%
Chen 2015	0.0%	0.0%	0.0%
Coffey 2018	0.0%	0.0%	0.0%	0.0%
Davies 2013	0.0%	0.0%	0.0%	0.0%	0.0%
Ellis 2018	0.0%	0.0%	0.0%	5.1%	0.0%	4.1%
Fleming 2009	0.0%	0.0%	0.0%	0.0%	0.0%	0.0%	0.0%
Foss 2016	0.0%	0.0%	0.7%	0.0%	0.0%	0.0%	0.0%	0.8%
Franklin 2018	0.0%	0.0%	0.0%	0.0%	0.0%	0.0%	0.0%	0.0%	1.5%
Frost 2014	0.0%	0.0%	0.0%	7.4%	0.0%	0.0%	0.0%	0.0%	2.9%	0.0%
Gorst 2019	0.0%	0.0%	0.0%	0.0%	0.0%	0.0%	1.7%	0.0%	0.0%	2.6%	0.0%
Graham-Rowe 2018	0.0%	0.0%	0.0%	0.0%	0.0%	0.0%	0.0%	0.0%	0.0%	0.0%	0.0%	0.0%
Ho 2010	0.0%	0.0%	0.0%	0.0%	0.0%	0.0%	0.0%	5.3%	1.6%	0.0%	6.9%	0.0%	0.0%
Horigan 2017	0.0%	0.0%	0.0%	0.0%	0.0%	0.0%	0.0%	0.0%	0.0%	0.0%	0.0%	0.0%	0.0%	0.0%
Inga-Britt 2018	0.0%	0.0%	0.0%	0.0%	0.0%	0.0%	0.0%	0.0%	8.4%	6.3%	5.4%	3.3%	0.0%	2.2%	0.0%
Jalil 2015	0.0%	0.0%	0.0%	0.0%	0.0%	0.0%	0.0%	0.0%	0.0%	0.0%	5.1%	0.0%	0.0%	0.0%	0.0%	0.0%
Jones 2017	0.0%	0.0%	0.0%	0.0%	0.0%	0.0%	1.7%	0.0%	0.0%	0.0%	0.0%	1.9%	0.0%	0.0%	0.0%	1.6%	0.0%
Kashim 2018	0.0%	0.0%	0.0%	0.0%	0.0%	0.0%	0.0%	0.0%	0.0%	0.0%	0.0%	0.0%	10.4%	0.0%	0.0%	0.0%	0.0%	0.0%
Kumar 2016	0.0%	0.0%	0.0%	0.0%	0.0%	0.0%	0.0%	14.3%	3.0%	0.0%	2.4%	2.2%	0.0%	7.1%	3.1%	0.0%	0.0%	0.0%	0.0%
Li 2014	0.0%	0.0%	0.0%	0.0%	0.0%	0.0%	0.0%	0.0%	0.0%	0.0%	0.0%	2.9%	0.0%	0.0%	0.0%	2.2%	0.0%	2.9%	0.0%	0.0%
Maine 2018	0.0%	0.0%	0.0%	0.0%	0.0%	0.0%	0.0%	0.0%	0.0%	0.0%	0.0%	0.0%	0.0%	0.0%	0.0%	0.0%	0.0%	0.0%	0.0%	0.0%	0.0%
Majeed-Ariss 2015	0.0%	0.0%	0.0%	0.0%	1.0%	0.0%	1.1%	3.0%	1.7%	1.4%	0.0%	3.8%	0.0%	3.1%	0.0%	0.0%	0.0%	9.1%	0.0%	6.8%	8.2%	0.0%
Matricciani 2015	0.0%	0.0%	0.0%	0.0%	2.0%	0.0%	0.0%	0.0%	0.0%	0.0%	0.0%	0.0%	0.0%	0.0%	0.0%	0.0%	0.0%	0.0%	0.0%	0.0%	0.0%	0.0%	0.0%
Miller 2018	0.0%	0.0%	0.0%	0.0%	0.0%	0.0%	0.0%	0.0%	0.0%	0.0%	2.9%	0.0%	0.0%	0.0%	0.0%	0.0%	0.0%	0.0%	0.0%	0.0%	0.0%	0.0%	0.0%	0.0%
Ng 2015	0.0%	0.0%	0.0%	0.0%	0.0%	0.0%	3.5%	0.0%	0.7%	0.0%	0.0%	0.0%	0.0%	0.0%	0.0%	0.0%	0.0%	0.0%	0.0%	0.0%	0.0%	0.0%	0.0%	0.0%	0.0%
Peimani 2018	0.0%	0.0%	0.0%	0.0%	0.0%	0.0%	0.0%	0.0%	0.0%	5.9%	0.0%	2.1%	0.0%	3.3%	0.0%	1.7%	0.0%	0.0%	0.0%	0.0%	3.3%	0.0%	2.6%	3.3%	0.0%	0.0%
Polinski 2013	0.0%	0.0%	0.0%	0.0%	0.0%	0.0%	4.8%	0.0%	0.0%	0.0%	0.0%	0.0%	0.0%	0.0%	0.0%	0.0%	0.0%	0.0%	0.0%	0.0%	0.0%	0.0%	0.0%	0.0%	0.0%	0.0%	0.0%
Psarou 2018	0.0%	0.0%	0.0%	0.0%	0.0%	0.0%	3.9%	7.1%	2.2%	0.0%	7.9%	7.1%	0.0%	3.7%	0.0%	1.8%	0.0%	2.2%	0.0%	11.1%	0.0%	0.0%	5.6%	0.0%	0.0%	4.8%	0.0%	0.0%
Pun 2009	0.0%	0.0%	0.0%	0.0%	0.0%	3.1%	2.0%	0.0%	0.0%	0.0%	0.0%	0.0%	0.0%	0.0%	0.0%	0.0%	0.0%	0.0%	0.0%	0.0%	0.0%	0.0%	0.0%	0.0%	0.0%	0.0%	0.0%	0.0%	0.0%
Rouyard 2017	0.0%	0.0%	0.0%	0.0%	0.0%	0.0%	0.0%	0.0%	0.0%	0.0%	0.0%	0.0%	0.0%	0.0%	0.0%	0.0%	0.0%	0.0%	0.0%	0.0%	0.0%	0.0%	1.4%	0.0%	0.0%	0.0%	0.0%	0.0%	0.0%	0.0%
Sarayani 2013	0.0%	0.0%	0.0%	0.0%	0.0%	0.0%	0.0%	0.0%	0.0%	0.0%	0.0%	0.0%	0.0%	0.0%	0.0%	0.0%	3.1%	0.0%	0.0%	0.0%	0.0%	0.0%	0.0%	0.0%	0.0%	0.0%	0.0%	0.0%	0.0%	0.0%	0.0%
Scarton 2014	0.0%	0.0%	0.0%	0.0%	2.1%	0.0%	0.0%	0.0%	0.0%	0.0%	0.0%	0.0%	0.0%	0.0%	0.0%	0.0%	0.0%	0.0%	0.0%	0.0%	0.0%	0.0%	1.6%	0.0%	0.0%	0.0%	0.0%	0.0%	0.0%	0.0%	0.0%	0.0%
Schmidt-Busby, 2018	0.0%	0.0%	0.0%	0.0%	0.0%	0.0%	0.0%	0.0%	0.7%	2.3%	0.0%	0.0%	0.0%	0.0%	0.0%	1.5%	0.0%	5.6%	0.0%	0.0%	0.0%	0.0%	0.0%	0.0%	2.3%	0.0%	2.0%	0.0%	0.0%	2.2%	0.0%	0.0%	0.0%
Singh 2016	0.0%	0.0%	0.0%	0.0%	0.0%	0.0%	0.0%	0.0%	0.0%	0.0%	0.0%	0.0%	0.0%	0.0%	0.0%	0.0%	0.0%	0.0%	0.0%	0.0%	0.0%	0.0%	0.0%	0.0%	0.0%	0.0%	0.0%	0.0%	0.0%	0.0%	0.0%	0.0%	0.0%	0.0%
Sohal 2015	0.0%	0.0%	0.0%	0.0%	0.0%	0.0%	0.0%	34.8%	1.5%	0.0%	0.0%	0.0%	0.0%	7.4%	0.0%	0.0%	0.0%	0.0%	0.0%	17.1%	0.0%	0.0%	8.5%	0.0%	0.0%	0.0%	2.4%	0.0%	5.4%	0.0%	0.0%	0.0%	0.0%	0.0%	0.0%
Spenceley 2006	0.0%	0.0%	0.0%	0.0%	0.0%	0.0%	0.0%	3.8%	0.0%	0.0%	2.7%	2.4%	0.0%	0.0%	0.0%	0.0%	0.0%	0.0%	0.0%	0.0%	4.2%	0.0%	2.8%	0.0%	0.0%	0.0%	0.0%	0.0%	0.0%	6.7%	0.0%	0.0%	0.0%	4.5%	0.0%	2.9%
Stiffler 2014	0.0%	0.0%	0.0%	3.7%	0.0%	0.0%	1.9%	0.0%	3.7%	2.9%	0.0%	4.4%	0.0%	0.0%	3.1%	9.4%	0.0%	0.0%	0.0%	2.4%	0.0%	0.0%	0.0%	0.0%	0.0%	0.0%	0.0%	0.0%	2.6%	0.0%	0.0%	0.0%	0.0%	2.0%	0.0%	0.0%	0.0%
Vanstone 2017	0.0%	1.6%	0.7%	0.0%	0.0%	0.0%	0.7%	0.0%	1.7%	1.5%	0.7%	1.4%	0.0%	0.8%	0.0%	4.0%	0.0%	7.3%	0.0%	0.7%	1.6%	0.0%	3.5%	0.0%	0.0%	0.0%	0.0%	0.0%	0.7%	0.7%	0.0%	0.0%	0.8%	2.7%	0.0%	0.0%	0.0%	0.7%
Verhoeven 2007	0.0%	0.0%	0.0%	0.0%	0.0%	0.0%	0.0%	0.0%	0.0%	0.0%	0.0%	0.0%	0.0%	0.0%	0.0%	0.0%	7.4%	0.0%	0.0%	0.0%	0.0%	0.0%	0.0%	0.0%	0.0%	0.0%	0.0%	0.0%	0.0%	0.0%	0.0%	0.0%	0.0%	0.0%	4.2%	0.0%	0.0%	0.0%	0.0%
Vongmany 2018	0.0%	0.0%	0.0%	0.0%	0.0%	0.0%	0.0%	0.0%	0.6%	0.0%	0.0%	3.1%	0.0%	0.0%	2.0%	2.7%	0.0%	9.8%	0.0%	1.7%	4.3%	0.0%	4.3%	0.0%	0.0%	0.0%	0.0%	0.0%	0.0%	0.0%	0.0%	0.0%	2.2%	6.1%	0.0%	0.0%	0.0%	1.7%	6.7%	0.0%
Zeh 2014	0.0%	0.0%	0.0%	0.0%	0.0%	0.0%	0.0%	24.1%	3.6%	0.0%	0.0%	0.0%	0.0%	3.0%	0.0%	1.6%	0.0%	2.0%	0.0%	21.1%	0.0%	0.0%	7.9%	0.0%	0.0%	0.0%	0.0%	0.0%	4.8%	0.0%	0.0%	0.0%	0.0%	0.0%	0.0%	25.0%	2.5%	0.0%	2.1%	0.0%	0.0%
Nam 2011	0.0%	0.0%	0.0%	0.0%	0.0%	0.0%	1.2%	0.0%	0.6%	0.0%	1.4%	1.4%	0.0%	0.0%	0.0%	0.0%	0.0%	0.0%	0.0%	0.0%	0.0%	0.0%	1.0%	0.0%	0.0%	1.4%	0.0%	1.7%	1.5%	3.2%	0.0%	0.0%	0.0%	1.3%	0.0%	0.0%	0.0%	0.0%	0.6%	0.0%	0.0%	0.0%
Wang 2012	0.0%	0.0%	0.0%	0.0%	0.0%	3.1%	0.0%	0.0%	0.0%	0.0%	0.0%	2.4%	0.0%	0.0%	0.0%	0.0%	0.0%	0.0%	0.0%	0.0%	0.0%	0.0%	2.8%	0.0%	0.0%	7.9%	0.0%	8.3%	6.1%	0.0%	0.0%	0.0%	0.0%	0.0%	0.0%	0.0%	0.0%	0.0%	0.0%	0.0%	0.0%	0.0%	3.2%
Albanese 2019	0.0%	2.9%	0.0%	0.0%	0.0%	0.0%	0.0%	0.0%	0.0%	0.0%	0.0%	0.0%	0.0%	0.0%	0.0%	1.5%	0.0%	1.8%	0.0%	0.0%	0.0%	0.0%	1.1%	0.0%	0.0%	0.0%	0.0%	0.0%	0.0%	2.2%	0.0%	0.0%	0.0%	0.0%	0.0%	0.0%	0.0%	0.0%	3.4%	0.0%	2.9%	0.0%	1.3%	0.0%
Jain 2020	0.0%	0.0%	0.0%	0.0%	0.0%	0.0%	0.0%	0.0%	0.0%	0.0%	0.0%	0.0%	0.0%	0.0%	0.0%	0.0%	0.0%	0.0%	0.0%	0.0%	0.0%	0.0%	0.0%	0.0%	0.0%	0.0%	0.0%	0.0%	0.0%	0.0%	0.0%	0.0%	0.0%	0.0%	0.0%	0.0%	0.0%	0.0%	0.0%	0.0%	0.0%	0.0%	0.0%	0.0%	0.0%
Joo 2021	0.0%	0.0%	0.0%	0.0%	0.0%	0.0%	0.0%	0.0%	0.0%	0.0%	0.0%	0.0%	0.0%	0.0%	0.0%	0.0%	0.0%	0.0%	0.0%	0.0%	0.0%	0.0%	0.0%	0.0%	0.0%	0.0%	0.0%	0.0%	0.0%	0.0%	0.0%	0.0%	0.0%	0.0%	0.0%	0.0%	0.0%	0.0%	0.0%	0.0%	0.0%	0.0%	0.0%	0.0%	0.0%	5.6%
Li-Geng2020	0.0%	0.0%	0.0%	0.0%	0.0%	0.0%	0.0%	0.0%	0.0%	0.0%	0.0%	5.9%	0.0%	0.0%	0.0%	0.0%	0.0%	15.6%	0.0%	0.0%	0.0%	0.0%	1.5%	0.0%	0.0%	0.0%	0.0%	0.0%	0.0%	0.0%	0.0%	0.0%	0.0%	0.0%	0.0%	0.0%	0.0%	0.0%	2.4%	0.0%	6.4%	0.0%	0.0%	0.0%	5.1%	0.0%	0.0%
Madsen 2019	0.0%	0.0%	0.0%	0.0%	0.0%	0.0%	0.0%	0.0%	0.0%	0.0%	0.0%	0.0%	0.0%	0.0%	0.0%	0.0%	0.0%	0.0%	0.0%	0.0%	0.0%	0.0%	0.0%	0.0%	0.0%	0.0%	0.0%	0.0%	0.0%	0.0%	0.0%	0.0%	0.0%	0.0%	0.0%	0.0%	0.0%	0.0%	0.0%	0.0%	0.0%	0.0%	0.0%	0.0%	0.0%	0.0%	0.0%	0.0%
Pennbrant 2020	0.0%	0.0%	0.0%	0.0%	0.0%	0.0%	0.0%	0.0%	0.0%	0.0%	0.0%	0.0%	1.3%	0.0%	0.0%	0.0%	0.0%	2.9%	0.0%	0.0%	0.0%	0.0%	0.0%	0.0%	0.0%	0.0%	0.0%	0.0%	0.0%	0.0%	0.0%	0.0%	0.0%	2.7%	0.0%	0.0%	0.0%	0.0%	0.8%	0.0%	0.0%	0.0%	0.0%	0.0%	0.0%	0.0%	0.0%	0.0%	0.0%
Rai 2020	0.0%	0.0%	0.0%	0.0%	0.0%	0.0%	1.9%	0.0%	0.0%	0.0%	0.0%	0.0%	0.0%	0.0%	0.0%	0.0%	0.0%	0.0%	0.0%	0.0%	0.0%	0.0%	0.0%	0.0%	0.0%	0.0%	0.0%	0.0%	0.0%	0.0%	0.0%	0.0%	0.0%	0.0%	0.0%	0.0%	0.0%	0.0%	0.0%	0.0%	0.0%	0.0%	0.0%	0.0%	0.0%	0.0%	0.0%	0.0%	0.0%	0.0%
Sibounheuang 2020	0.0%	0.0%	0.0%	0.0%	0.0%	0.0%	0.0%	0.0%	0.0%	8.8%	0.0%	6.5%	0.0%	0.0%	0.0%	5.3%	0.0%	2.0%	0.0%	0.0%	3.2%	0.0%	2.6%	0.0%	0.0%	2.1%	2.3%	0.0%	0.0%	0.0%	0.0%	0.0%	0.0%	1.9%	0.0%	0.0%	0.0%	0.0%	0.0%	0.0%	1.6%	0.0%	1.4%	0.0%	0.0%	0.0%	0.0%	0.0%	0.0%	0.0%	0.0%
Suglo 2020	0.0%	0.0%	0.0%	0.0%	3.6%	0.0%	0.0%	0.0%	0.0%	0.0%	0.0%	0.0%	0.0%	0.0%	0.0%	0.0%	0.0%	0.0%	0.0%	0.0%	0.0%	0.0%	0.0%	0.0%	0.0%	0.0%	0.0%	0.0%	0.0%	0.0%	0.0%	0.0%	0.0%	0.0%	0.0%	0.0%	0.0%	0.0%	0.0%	0.0%	1.8%	0.0%	0.0%	0.0%	0.0%	0.0%	0.0%	0.0%	0.0%	0.0%	0.0%	0.0%
Walker 2019	0.0%	0.0%	0.0%	0.0%	0.0%	0.0%	0.0%	0.0%	0.0%	0.0%	0.0%	0.0%	0.0%	0.0%	0.0%	0.0%	0.0%	0.0%	0.0%	0.0%	0.0%	0.0%	0.0%	0.0%	0.0%	0.0%	0.0%	0.0%	0.0%	0.0%	0.0%	0.0%	0.0%	0.0%	0.0%	0.0%	0.0%	0.0%	0.0%	0.0%	0.0%	0.0%	0.0%	0.0%	0.0%	0.0%	0.0%	0.0%	0.0%	0.0%	0.0%	0.0%	0.0%
